# Structural characterization of antibiotic self-immunity tRNA synthetase in plant tumour biocontrol agent

**DOI:** 10.1038/ncomms12928

**Published:** 2016-10-07

**Authors:** Shaileja Chopra, Andrés Palencia, Cornelia Virus, Sarah Schulwitz, Brenda R. Temple, Stephen Cusack, John Reader

**Affiliations:** 1 Department of Cell Biology and Physiology, The University of North Carolina at Chapel Hill, Chapel Hill, North Carolina 27599-7090, USA; 2 European Molecular Biology Laboratory, Grenoble Outstation and Unit of Virus Host-Cell Interactions, UJF-EMBL-CNRS, UMI 3265, 71, Avenue des Martyrs, CS 90181, 38042 Grenoble Cedex 96 rue Jules Horowitz, BP181, Grenoble Cedex 9 38042, France; 3 R.L. Juliano Structural Bioinformatics Core Facility, The University of North Carolina at Chapel Hill, Chapel Hill, North Carolina 27599, USA

## Abstract

Antibiotic-producing microbes evolved self-resistance mechanisms to avoid suicide. The biocontrol *Agrobacterium radiobacter* K84 secretes the Trojan Horse antibiotic agrocin 84 that is selectively transported into the plant pathogen *A. tumefaciens* and processed into the toxin TM84. We previously showed that TM84 employs a unique tRNA-dependent mechanism to inhibit leucyl-tRNA synthetase (LeuRS), while the TM84-producer prevents self-poisoning by expressing a resistant LeuRS AgnB2. We now identify a mechanism by which the antibiotic-producing microbe resists its own toxin. Using a combination of structural, biochemical and biophysical approaches, we show that AgnB2 evolved structural changes so as to resist the antibiotic by eliminating the tRNA-dependence of TM84 binding. Mutagenesis of key resistance determinants results in mutants adopting an antibiotic-sensitive phenotype. This study illuminates the evolution of resistance in self-immunity genes and provides mechanistic insights into a fascinating tRNA-dependent antibiotic with applications for the development of anti-infectives and the prevention of biocontrol emasculation.

## Abstract (web summary)

The bacterium *Agrobacterium radiobacter* K84 secretes an antibiotic that is transported into the plant pathogen *A. tumefaciens* and processed into the toxin TM84. Here, the authors identify a mechanism whereby the antibiotic-producing microbe resists its own toxin.

Microbes that produce antimicrobial agents often prevent cell suicide by co-expressing self-immunity proteins that employ a variety of different resistance mechanisms^1^. Genes encoding self-immunity proteins can also act as a source of antibiotic resistance in the environment when they are horizontally transferred to microbial pathogens^2^. This threatens not only the successful treatment of human pathogens but also prevention of important animal and crop diseases^3^. One mechanism of self-immunity is to encode resistant variants of the enzyme targeted by the antibiotic. A fascinating example of such an antibiotic-producing organism is *Agrobacterium radiobacter* strain K84, which is used as a biocontrol agent of the plant pathogen *A. tumefaciens* that causes plant tumours in a number of agriculturally important species^4^,^5^,^6^. The biocontrol produces the ‘Trojan Horse' antibiotic agrocin 84 (refs 7, 8, 9) that mimics a plant tumour-derived substrate to gain entry by subterfuge into the pathogen^10^,^11^, where it is processed into a toxin termed TM84 (refs 10, 12) (Figs 1 and 2a). We recently identified the molecular target of TM84 in *A. tumefaciens* as leucyl-tRNA synthetase (LeuRS_At_) which catalyses an essential first step in protein synthesis^12^. To prevent cell suicide, *A. radiobacter* K84 contains a second, non-essential LeuRS, termed AgnB2, which is resistant to TM84 (refs 12, 13, 14) (Fig. 1). The *agnB2* gene is encoded on the 44 kb plasmid pAgK84, along with all the genes required to biosynthesize agrocin 84 (refs 14, 15). Importantly, horizontal gene transfer of the pAgK84 plasmid from *A. radiobacter* K84 to the pathogen has been previously reported to occur in the field, resulting in agrocin 84-resistant strains of *A. tumefaciens* encoding the *agnB2* gene^5^,^16^,^17^. Thus, understanding the molecular mechanisms by which AgnB2 provides immunity to agrocin 84 is crucial for combating antibiotic resistance.

The molecular target of TM84, LeuRS_At_, is an essential enzyme that specifically ligates leucine to its cognate tRNA^Leu^ isoacceptors, a critical step before protein synthesis at the ribosome. The aminoacylation of tRNA^Leu^ is catalysed in a two-step mechanism in all LeuRSs: in the tRNA-independent first step, leucine is activated by ATP to form the essential obligate intermediate leucyl-adenylate (Leu-AMP) that is stably bound to the enzyme. In the second step, a bound tRNA^Leu^ repositions its CCA 3′-end into the LeuRS active site where activated-leucine is transferred to the tRNA. The essential catalytic nature of LeuRSs, and the 19 other canonical aminoacyl-tRNA synthetases (AARSs), has led to these enzymes emerging as prominent targets for anti-pathogens^18^,^19^, ranging in the prevention of fungal diseases in humans^20^ to bacterial plant pathogens^12^. A substantial amount of work has been performed on stable analogues of aminoacyl-adenylates that bind tightly to an AARS active site, and preventing activation of the amino acid with ATP^21^,^22^. However, these molecules often cannot be transported efficiently into cells and can lack discrimination between microorganism and the intended host^18^.

We recently showed that TM84 does not behave like other stable aminoacyl-adenylate analogues but, instead, employs a unique tRNA-dependent inhibition mechanism^23^,^24^. TM84 binds only weakly to the LeuRS-active site on its own and requires the presence of tRNA^Leu^ to form a tight binding ternary inhibition complex^23^. The X-ray crystal structure of the *Escherichia coli* LeuRS_Ec_·tRNA^Leu^·TM84 ternary complex shows TM84 bound in the Leu-AMP-binding site of the catalytic domain. The tRNA^Leu^ is bound to LeuRS in an ‘aminoacylation-like' conformation with the CCA 3′-end of the tRNA^Leu^ entering into the active site with the terminal adenosine hydrogen bonding (H-bonding) directly with TM84 (ref. 23). The positioning of the tRNA^Leu^ acceptor stem is accompanied with a substantial movement of the catalytic K^619^MSK^622^S motif. The K619 sidechain bonds to the penultimate tRNA^Leu^ cytidine base, while K622 extends across the adenylate analogue and interacts with the phosphate of TM84 via a non-bridging oxygen. The repositioning of the Lys-Met-Ser-Lys-Ser (KMSKS) loop and tRNA^Leu^ combine to effectively bury the bound TM84, thus explaining how the presence of tRNA^Leu^ induces the tight binding of LeuRSs to TM84.

AgnB2 exhibits a high degree of resistance to TM84 (ref. 12) despite the close similarity of TM84 to Leu-AMP (Fig. 2a) and the need for AgnB2 to adopt a true aminoacylation complex as exhibited in a recent LeuRS_Ec_·tRNA^Leu^·Leu-AMS crystal structure^25^. These observations raise the question of how does AgnB2 achieve resistance to TM84 without dramatically interfering with the catalysis of the aminoacylation reaction. This is particularly intriguing as primary sequence alignments of AgnB2 and TM84-sensitive LeuRSs show a high similarity of residues, including catalytic motifs (except a single Q instead of M at the KMSKS motif). Here, we examine the TM84 resistance mechanisms employed by AgnB2 using both thermodynamics and steady-state enzyme kinetics. We also present the X-ray crystal structure of AgnB2 in complex with tRNA^Leu^ and a stable leucyl-adenylate analogue and reveal tertiary interactions unique to AgnB2. We finally identify key TM84 immunity determinants in AgnB2 and the TM84-sensitive LeuRS_At_ and show that it is possible to partially inter-convert the TM84 sensitivity properties of these enzymes.

## Results

### Analysis of AgnB2 steady state enzyme kinetics

**Figure 2:**
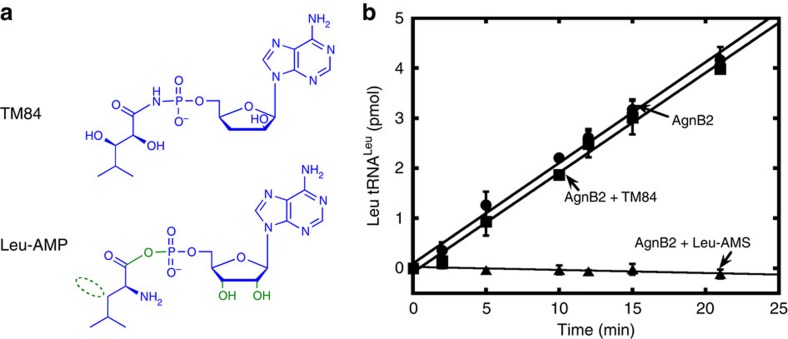
AgnB2 discriminates between a stable Leu-AMP analogue and TM84. (**a**) Chemical structures of Leu-AMP and TM84 with differences highlighted in green. A non-hydrolysable adenylate analogue called Leu-AMS, which contains a N-sulfamoyl linkage in place of the phosphoanhydride of Leu-AMP, was used in biophysical and crystallography experiments. (**b**) Effect of TM84 and Leu-AMS on the aminoacylation reaction of wt AgnB2. Aminoacylation reactions were carried out using 2 nM wt AgnB2 only (●) and wt AgnB2 in the presence of 1 μM TM84 (■) and 20 nM Leu-AMS (▲) at 28 °C, pH 7.4 and initiated using 1 mM ATP. Error bars represent s.d. (*n*=3).

The ability of AgnB2 to discriminate between TM84 and the obligate reaction intermediate Leu-AMP prompted us to examine differences between the two molecules. As shown in Fig. 2a, TM84 contains a stable N-acyl phosphoamidate bond, instead of the reactive phosphoanhydride linkage in Leu-AMP. In addition, TM84 has a deoxy-arabinose sugar group, instead of a ribose with subsequent loss of a 3′-hydroxyl (OH) and a repositioned 2′-OH above the sugar ring, and replacement of the primary amine with another OH group. For TM84-sensitive LeuRSs, these subtle differences between toxin and reaction intermediate allow TM84 to employ a tRNA-dependent inhibition mechanism where the CCA 3′-end of the tRNA^Leu^ binds in an ‘aminoacylation-like conformation' into the active site of the enzyme and directly hydrogen bonds with the antibiotic^23^. The ‘aminoacylation-like' conformation of tRNA in the LeuRS_Ec_·tRNA^Leu^·TM84 ternary inhibition complex exhibits a high degree of similarity with the tRNA^Leu^ in the true LeuRS aminoacylation conformation^25^. Since the tRNA^Leu^ bound to AgnB2 still has to reposition its CCA 3′-end into the active site during catalysis of the aminoacyl transfer reaction, we questioned whether destabilization of the TM84 ternary complex in AgnB2 might be reflected in the kinetic properties of AgnB2.

Our steady-state kinetic analysis of AgnB2 examined both the overall aminoacylation reaction and the first step, the amino acid activation reaction, using the ATP/PPi exchange reaction. We firstly determined the apparent TM84 inhibition constant (*K_i_*) for AgnB2 in the aminoacylation reaction by altering the ATP concentrations of the substrate and pre-incubating the AgnB2·tRNA^Leu^ complex before initiating the reaction. The data revealed an apparent *K_i_* of 4.72±0.75 μM (Supplementary Fig. 1). We then determined the apparent TM84 *K_i_* for AgnB2 by altering the leucine concentration and keeping the ATP fixed giving a value of 1.38±0.28 μM (Supplementary Fig. 1a,b). Both values are more than three orders of magnitude weaker than the *K*_*i*_^app^ determined previously for the natural target LeuRS_At_ (ref. 23). Importantly, TM84 acted as a weak competitive inhibitor of AgnB2 with regards to both ATP and leucine (Supplementary Fig. 1a,b, respectively) and therefore still binds to the synthetic active site and not at an alternative site. We also found that AgnB2 is resistant to TM84, but is still potently inhibited by Leu-AMS (a non-hydrolysable analogue of the Leu-AMP reaction intermediate), for both the amino acid activation (Supplementary Fig. 2) and overall aminoacylation reactions (Fig. 2b). Substantial weakening of the binding of the aminoacyl adenylate to AgnB2 did not evolve as a solution to generating resistance to TM84, as this would severely affect the catalysis of amino acid activation.

As shown in Table 1, AgnB2 and LeuRS_At_ share similar *k*_cat_ values for all substrates. The higher *K*_m_ for tRNA^Leu^ in AgnB2 may reflect an alteration in the interactions of AgnB2 with tRNA^Leu^ that is relevant to resistance to the tRNA-dependent TM84 inhibitor. The *K*_m_ for ATP is a small 3.8-fold higher affinity value for AgnB2 than for LeuRS_At_, while the leucine affinities show minimal differences. In the case of ATP/PPi exchange reaction, AgnB2 showed a higher *K*_m_ for ATP compared with the aminoacylation reaction (10-fold increase). Interestingly, these kinetic comparisons represent only small energetic differences between the two enzymes, yet still lead to enzymes with pronounced differences in TM84 sensitivity. In addition, the steady-state kinetic properties shows that the ATP saturation plot for the overall aminoacylation reaction shows a degree of substrate inhibition, whereas the ATP/PPi exchange shows no substrate inhibition for ATP (data not shown) when tRNA^Leu^ is absent. One possibility is the population of a second unproductive ATP-binding site in the AgnB2 active site that may in part explain the differences between the apparent TM84 *K_i_*'s determined for AgnB2 when varying leucine and ATP (Supplementary Fig. 1). Interestingly, we do not observe any ATP substrate inhibition for LeuRS_At_. The data suggest that different substrate-dependent conformations populated by AgnB2 may be important for TM84 resistance.

### tRNA^Leu^ has reduced effect on the affinity of TM84 to AgnB2

If resistance of AgnB2 is based on abolishing of the normal tRNA-dependent inhibition mechanism employed by TM84 (ref. 23) then one would expect to see a decrease in the affinity for TM84, tRNA^Leu^ and/or for TM84 in the presence of tRNA or vice versa. To test these possibilities, we used isothermal calorimetry (ITC) (Supplementary Table 1) to investigate the ligand binding modes to AgnB2 (ref. 23). *K*_d_ values of 1052, nM for TM84 and 310 nM for tRNA were obtained, indicating reductions in AgnB2-binding affinities for both TM84 (6.9-fold) and tRNA (3.7-fold), compared with LeuRS_At_ (ref. 23). Further examination of TM84 binding to AgnB2 (with pre-bound tRNA) showed a *K*_d_ of 332 nM, which suggests a substantial destabilization of the ternary complex (AgnB2·tRNA·TM84) (∼330-fold decrease) as compared with the LeuRS_At_·tRNA·TM84 complex (Supplementary Table 2). Interestingly, we also see no evidence of a binary isotherm for TM84 binding to AgnB2·tRNA^Leu^ species, unlike LeuRS_At_. Thus, AgnB2 has a significantly weakened affinity for TM84 as compared with LeuRS_At_. Since TM84 and the Leu-AMP analogue bind to the same active site but potentially to different conformations, we examined if AgnB2-binding affinity for Leu-AMS had been substantially weakened. Our results show that Leu-AMS binds to AgnB2 with low nanomolar binding affinity (*K*_d_=4.4 nM), which is slightly weaker than to LeuRS_At_ (1.6 nM). The fact that AgnB2 binds tightly to Leu-AMS is not so surprising since, firstly, AgnB2 has highly conserved residues at the synthetic site compared with other LeuRSs and, secondly, the enzyme must bind tightly to the Leu-AMP intermediate to catalyze the aminoacylation reaction. This result was supported by our enzyme kinetic observations above which showed that Leu-AMS is a low nanomolar inhibitor of both the AgnB2-catalyzed aminoacylation and amino acid activation reactions, demonstrating that the analogue efficiently competes with ATP and leucine substrates (Supplementary Fig. 1).

Our thermodynamic results suggest that AgnB2 may achieve resistance to TM84 by not simply weakening the affinity for TM84 (and/or tRNA^Leu^) but most significantly by destabilizing the formation of the AgnB2·tRNA^Leu^·TM84 complex. Interestingly, this is accomplished with only minimal change to affinity for the adenylate, allowing the enzyme to still efficiently carry out aminoacylation.

### Structure of AgnB2·tRNA^Leu^·Leu-AMS ternary complex

**Figure 3:**
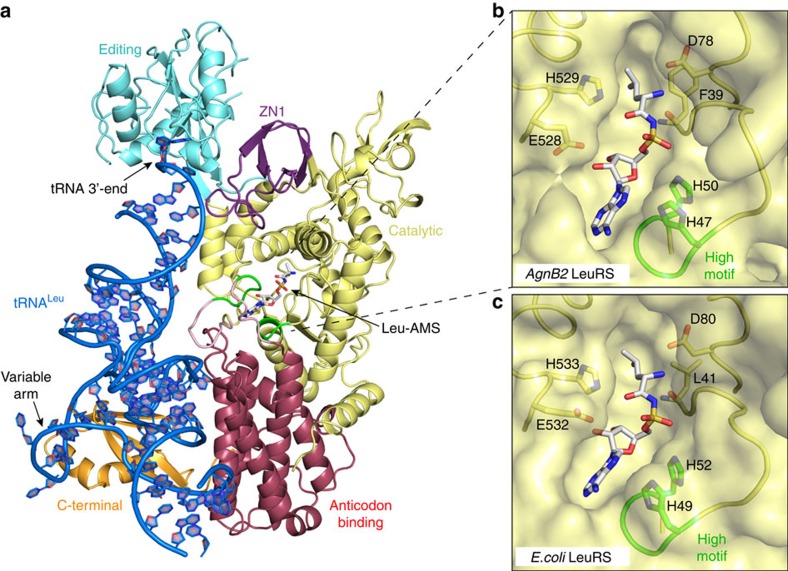
Structure of the AgnB2·tRNA^Leu^·Leu-AMS complex. (**a**) X-ray structure of wild-type AgnB2·tRNA^Leu^·Leu-AMS complex with 3′-tRNA^Leu^ (blue) bound in the editing site (PDB Code—5AH5). The catalytic, editing, anticodon binding and C-terminal domains are displayed in yellow, cyan blue, red and gold, respectively. Leu-AMS bound to the synthetic active site is represented in a stick structure and the catalytic KQSKS and HIGH loops are highlighted in green. (**b**,**c**) Comparison of wt AgnB2·tRNA·Leu-AMS complex and LeuRS_Ec_· tRNA^Leu^·Leu-AMS complex binding site residues. The surface of the catalytic active site of the protein is depicted in yellow with Leu-AMS bound (stick structure).

To identify structural interactions in AgnB2 that may contribute to resistance, we performed extensive crystallizations trials with AgnB2, agrobacterium tRNA^Leu^ and multiple combinations of substrates or inhibitors. We obtained an X-ray crystal structure of AgnB2 in complex with tRNA^Leu^ and Leu-AMS; the final model was refined to a final R-factor of 19.4% (R-free=23.8%) and a final resolution of 2.1 Å (Table 2, Fig. 3a and Supplementary Fig. 3).

**Figure 4:**
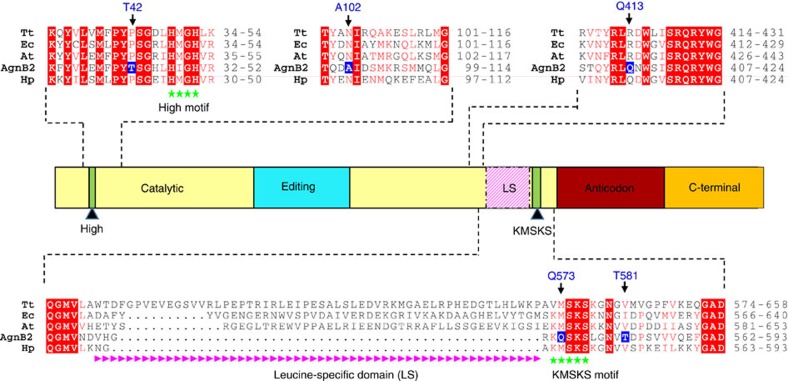
Domain structure and primary sequence alignment of LeuRSs and AgnB2. Domain structure of LeuRS and primary sequence alignment of the catalytic domain and LS domains of *Thermus thermophilus* LeuRS (LeuRS_Tt_), LeuRS_Ec_, LeuRS_At_, plasmid encoded AgnB2 (from *A. radiobacter* strain K84) and LeuRS_Hp_. The highly conserved regions are depicted in grey and the residues highlighted in bold (blue) represent the non-conserved residues in AgnB2. In addition, both AgnB2 and LeuRS_Hp_ lack the leucine-specific domain (pink) that is present in other LeuRSs.

The overall AgnB2·tRNA^Leu^·Leu-AMS structure (Fig. 3a, and see domain architecture in Fig. 4) presents a similar conformation to a number of bacterial LeuRS structures obtained in complex with tRNAs^Leu^, in which the CCA 3′-end is positioned into the editing active site and represents what appears to be the preferred LeuRS conformation^20^,^25^,^26^. This editing conformation of the tRNA in the AgnB2 complex is in contrast to the structures of the TM84 ternary inhibition complex of LeuRS_Ec_·tRNA^Leu^·TM84 and the LeuRS_Ec_·tRNA^Leu^·Leu-AMS aminoacylation complex which have the CCA 3′-end penetrating into the catalytic active site of the enzyme positioned next to the bound inhibitors^23^,^25^. Another important interaction observed in the structure is the C-terminal domain interaction with the variable loop of *Agrobacterium* tRNA^Leu^ (Supplementary Fig. 4a). A comparison of the aminoacylation and editing states in LeuRS_Ec_, shows the C-term domain coupled to the variable arm of *E. coli* tRNA^Leu^ rotates significantly upwards towards the catalytic domain in the aminoacylation state. It has been hypothesized that this C-term domain movement propagates towards the main body of the tRNA^Leu^ and towards the acceptor stem, and together with rotations of other external domains, or residues, forces the 3′-end of the tRNA into the catalytic site. Interestingly, the tRNA^Leu^ from *Agrobacterium* has a shorter variable stem than the *E. coli* tRNA, therefore questioning whether this propagation force due to coupling of tRNA with the C-term domain is also required in AgnB2 for aminoacylation. In fact, a comparison of the C-terminal domain from AgnB2 compared with the *E. coli* editing structure (LeuRS_Ec_·tRNA^Leu^)^25^ shows there is a rotation of about 18.5° and the shorter variable loop of agrobacterium only makes one contact with the phosphate of nucleotide 46f (P46f) vs the *E. coli* tRNA which makes multiple contacts with the variable domain^25^ (Supplementary Fig. 4a,b). This structure may give new insights into the function of this stem-loop.

### Unique amino acid substitutions in the AgnB2 catalytic site

Close inspection of the Leu-AMS-binding site in the AgnB2 structure reveals that the residues at the catalytic site are very similar compared with those found in LeuRS_**Ec**_ Fig. 3b,c. There are small differences including F39 in AgnB2, forming part of the hydrophobic pocket for the leucine, vs the corresponding L41 found in the LeuRS_Ec_. Additionally, E528 in AgnB2 exhibits a different rotamer away from ribose hydroxyl vs the corresponding E532 residue in the LeuRS_**Ec**_ aminoacylation complex, which is situated more closely to the ribose hydroxyl. The LeuRS_**Ec**_ E532 plays an important role in catalysis of the aminoacylation reaction, as suggested by the LeuRS_Ec_·tRNA^Leu^·Leu-AMS complex structure and confirmed by biochemical experiments^25^. The observation that TM84-sensitive LeuRS_Ec_ and resistant AgnB2 showed no substantial differences between active site residues directly interacting with Leu-AMS might explain how the two enzymes can bind equally well to Leu-AMS and carry out aminoacylation with close kinetic parameters (see above). It therefore follows that no AgnB2 residues are directly in a position to interrogate the 5′ N-acyl phosphoramidate bond of TM84 when it is bound to the enzyme alone. The weaker binding of TM84 to AgnB2, with or without tRNA^Leu^, compared with LeuRS_At_ might be explained by residues or domains that do not directly interact with the adenylate analogue, but which could promote long range effects or conformational changes affecting the catalytic site or perturbing the interaction with tRNA^Leu^.

**Figure 5:**
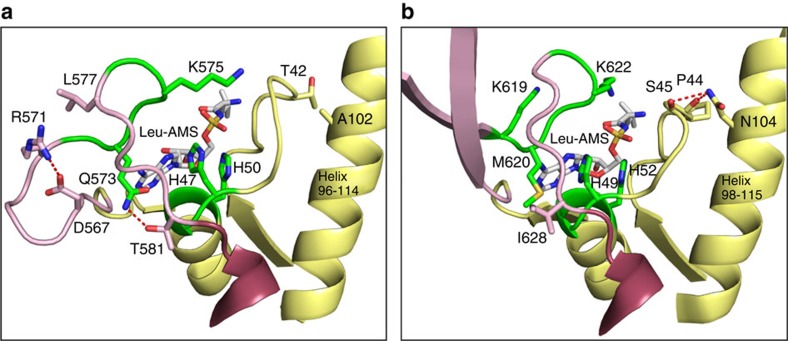
Interactions of key residues in the catalytic active site of AgnB2. Ribbon structure of the catalytic active site of (**a**) AgnB2·tRNA^Leu^·Leu-AMS complex and (**b**) *E. coli*·tRNA^Leu^·Leu-AMS complex. AgnB2 key residues interacting with Leu-AMS (shown as white sticks) or adjacent to the catalytic site are depicted as sticks with the following colour code: residues of the K^572^QSKS^576^ and of the H^47^IGH^50^ catalytic motifs, are coloured in green; core permissive residues T42 and A102 are shown in yellow; T581 which H-bonds to Q573 in pink; and D567 and R571 which forms the salt bridge mutated in this study and residue L577 are also in pink. *E. coli* LeuRS equivalent residues in the aminoacylation complex (PDB: 4AQ7) are shown in panel **b**. Key interactions in both the AgnB2 and the *E. coli* complexes are shown as red-dashed lines.

The KMSKS catalytic peptide motif, along with the HIGH loop, is present in all Class I aaRSs, including LeuRSs, and has been shown to play a critical role in the both the amino acid activation reaction and the aminoacyl transfer reactions^27^,^28^. Biochemical and biophysical studies on class I synthetases, including LeuRS, shows that ATP binding to the catalytic active site leads to a conformational change in the mobile KMSKS loop shifting it from an ‘open' to a ‘closed' conformation with the two lysine residues of the KMSKS loop interacting with the α and β phosphates of the ATP^27^,^28^,^29^. Once the aminoacyl-adenylate is formed, the KMSKS loop moves into a ‘semi-open' conformation to allow for the entry of the CCA 3′-end of the tRNA into the active site. Finally, once the CCA 3′-end of the tRNA is in the active site, the KMSKS loop once again adopts the closed conformation^25^,^27^,^30^. In the case of AgnB2, the highly conserved methionine of the KMSKS motif is interestingly replaced by a glutamine residue (Q573). Our structure shows that Leu-AMS is bound in the active site with the mobile KQSKS loop present in a semi-open conformation (Supplementary Fig. 5). This loop position is in contrast to the fully closed position exhibited by the KMSKS loop in the LeuRS_Ec_·tRNA·TM84 ternary complex where the second lysine of the KMSKS loop (K622) directly interacts with the non-bridging phosphate of the nucleotide analogue and the first lysine of the KMSKS loop (K619) interacts with cytosine 75 of the tRNA 3′-end^23^. In Class I aaRSs, this conserved methionine forms a number of hydrophobic packing interactions and is thought to play an important role in aminoacylation^31^,^32^,^33^,^34^. In LeuRS structures it also forms a main chain interaction with the adenine ring of the bound adenylate^25^,^35^. The equivalent position in AgnB2, Q573, forms a hydrogen bond side-chain interaction with the side chain of T581 (Fig. 5a), which is found to be a conserved hydrophobic residue in TM84 sensitive LeuRSs (I628 in LeuRS_Ec_) (Fig. 5b). It is interesting to note that the AgnB2 KQSKS loop exhibits a distinctly shifted position relative to the Leu-AMP binding pocket (Supplementary Fig. 5), perhaps due to these changes. The fact that a glutamine residue is found in this critical catalytic motif but not in any other LeuRS in nature, may indicate an important role for Q573 in TM84 resistance. It maybe that a substantial number of compensatory mutations are required to accommodate Q573 in AgnB2.

T42 and A102 are two other residues that are specific to AgnB2. T42 is positioned upstream from the catalytic H^47^IGH^50^ motif, while the side chain of A102 is situated in close proximity to T42 on a neighbouring α-helix (Fig. 5). Importantly, the presence of A102 in AgnB2, instead of an asparagine as found in the LeuRS_Ec_ structure (N104), leads to loss of an H-bond interaction between the α-helix (96–114) and a main-chain peptide bond located at T42 (P45 LeuRS_Ec_). Both these modifications could allow for subtle changes in the positioning of the neighbouring HIGH motif that binds TM84.

### AgnB2 has modified tRNA elements compared with LeuRS_Ec_

A prominent feature of the AgnB2 structure is the lack of an idiosyncratic leucyl-specific insertion domain (LS-domain), which is present in bacterial LeuRS, except in a few cases such, as *Bacillus subtilis* or *Mycoplasma mobile*^25^,^36^, and as seen in the *Helicobacter pylori* LeuRS (LeuRS_Hp_) primary amino acid sequence in Fig. 4. The LS-domain undergoes substantial movement between the LeuRS_Ec_ editing, and the TM84 and Leu-AMS bound ‘aminoacylation-like' conformations, buttressing the highly mobile KMSKS loop as well as guiding the tRNA into the catalytic site, as found in the aminoacylation conformation^25^. The missing LS-domain in AgnB2 is replaced by a small connecting loop that contains a salt bridge between residues D567 and R571. While the LS domain was shown not to be absolutely required for the aminoacylation or editing reactions of LeuRS *per se*^37^, it has been shown to be important for tRNA binding and is able to distinguish between the isoacceptors of tRNA^Leu^ in *E. coli*^38^. Our thermodynamic studies showed a ∼3.5-fold reduction in tRNA^Leu^ affinity between LeuRS_At_ and AgnB2 (Supplementary Tables 1 and 2), raising the possibility that the modification of tRNA interacting elements in AgnB2 may play a role in TM84 resistance (tested below).

It has been reported that residues distant from the active site can also be crucial in forming interactions with the negatively charged backbone of the tRNA^Leu^ CCA 3′-end and its repositioning in catalysis^38^. Mutation of the residue R418 to a neutral amino acid, such as glutamine, decreased the aminoacylation activity in LeuRS_Ec_ by reduction of the residence of the 3′-end in the enzyme active site^37^,^38^. Importantly, the AgnB2 structure shows the equivalent residue to R418 in LeuRS_Ec_ is the neutrally charged Q413 (Fig. 4). Thus, it is possible that Q413 in AgnB2 has a negative effect on binding of the tRNA-dependent inhibitor TM84.

### Identification of permissive mutations in the catalytic core

Having identified possible TM84 sensitivity/resistance determinants by comparison of the AgnB2 structure with other LeuRSs known to be sensitive to the toxin, we made mutants of AgnB2 and also LeuRS_At_ to test the contribution of these sites to antibiotic resistance. Because of the limited number of possible mutants and permutations that could be constructed using this approach, not all potential TM84 resistance/sensitivity determinants could be tested. We initially assayed candidate mutants using a biological assay (see Methods), followed by *in vitro* enzyme inhibition experiments combined with assessment of direct binding by calorimetry on promising constructs.

**Figure 6:**
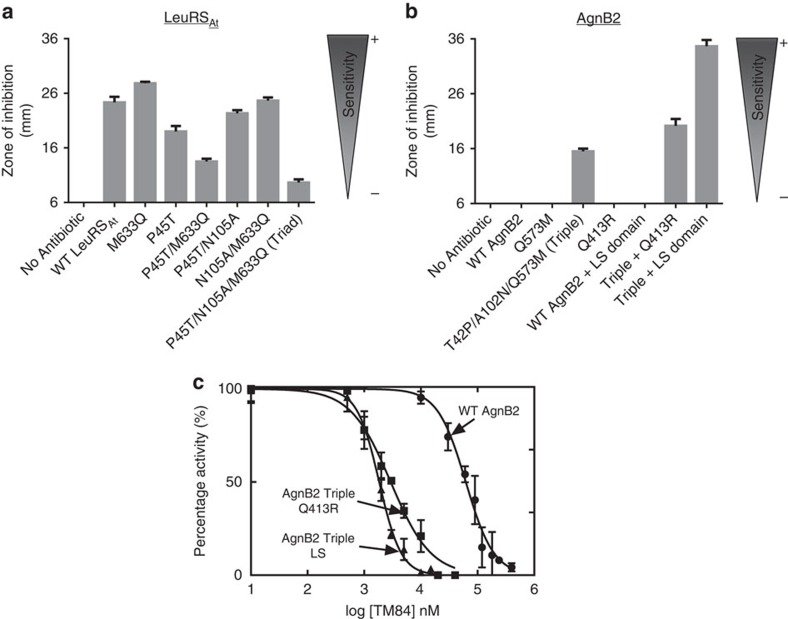
Comparison of antibiotic sensitivity of wt and mutant forms of LeuRS_At_ and AgnB2. *A. tumefaciens* NTL4 T7*pol*-Gm-*FRT* (pYW15d-*acs ΔaccR*) strain transformed with pBBR1MCS-3 plasmids containing genes encoding wt or mutant LeuRS_At_ or AgnB2 constructs were tested for their sensitivity towards 50 μl of agrocin 84 (60 μM) placed in central well of a bioassay plate. For negative control, sterile ddH_2_O was used instead of the antibiotic. (**a**) Bioassay results in graphical form indicating the sensitivity of wt LeuRS_At_ and LeuRS_At_ mutants; and (**b**) the resistance properties of wt AgnB2 and selected AgnB2 mutants; (**c**) Comparison of the TM84 inhibition of the aminoacylation reactions of wt AgnB2 (●), AgnB2 Triple Q413R mutant (■) and AgnB2 Triple LS domain mutant (▲). Dose–response curves and the resulting IC_50_ values were obtained at saturating leucine and (0.675 mM) ATP concentrations (see Methods). Error bars represent s.d. from three independent experiments.

Three of the putative resistance determinants in AgnB2, Q573, A102 and T42 described above (Fig. 5a), reside in the catalytic domain and differ from the highly conserved residues in TM84-sensitive LeuRSs. We introduced these mutations at corresponding positions (as determined by homology modelling) in the TM84-sensitive enzyme LeuRS_At_ (P45T, N105A and M633Q) and tested whether these mutations imparted resistance using our *in vivo* assay (Fig. 6a). The single M633Q mutation in the K(M/Q)SKS loop is restrictive in nature, as it was not tolerated in the LeuRS_At_ enzyme, most likely resulting in a functionally inactive enzyme. In contrast, the P45T (Fig. 6a) or N105A (data not shown) are permissive mutations and had a small effect on enzyme function or TM84 sensitivity. A P45T/N105A double mutant showed no increase in resistance for LeuRS_At_. When the P45T was introduced with M633Q in a double mutant, the P45T acted as a permissive mutation, increasing the resistance of the mutant enzyme. Finally, when all three mutations were introduced into LeuRS_At_ (P45T/N105A/M633Q, termed LeuRS_At_ Triad) there was a significant increase in resistance to the antibiotic (Fig. 6a) compared with wild type (wt).

Kinetic inhibition experiments on LeuRS_At_ Triad showed the enzyme was resistant to to concentrations of TM84 up to 500 nM (Supplementary Fig. 6a). This is in contrast to wt LeuRS_At_, which is sensitive to very low concentrations of TM84 (*K*_i_^app^=0.3±0.1 nM)^23^. The introduction of the Triad mutations also negatively affected the percentage of active enzyme, presumably because these modified residues not only alter engagement of the enzyme with the antibiotic but also modify the interaction of the active site with its substrates. Subsequent thermodynamic analysis of the binding of TM84 to the LeuRS_At_ Triad alone and the LeuRS_At_ Triad pre-incubated with tRNA^Leu^ showed respective 10-fold and 28-fold decreases in binding affinity, as compared with LeuRS_At_ (Supplementary Table 2).

To see if the three reverse mutations in AgnB2 could make the enzyme more sensitive to TM84, a variant containing all three mutations (T42P/A102N/Q573M termed AgnB2 Triple) was constructed. The AgnB2 Triple showed a distinct increase in TM84 sensitivity (larger clearance zone) as detected by bioassay (Fig. 6b). An individual, Q573M mutant did not increase TM84 sensitivity of AgnB2 (Fig. 6b). Kinetic analysis of the AgnB2 Triple mutant showed a 1.8-fold increase in resistance using a fixed concentration of ligand (Supplementary Fig. 6b). Thermodynamic analysis of the binding of TM84 to the AgnB2 Triple alone showed no significant effect on TM84 binding, while there was a 3.5-fold increase in binding of TM84 to the AgnB2·tRNA^Leu^ complex (Supplementary Table 1).

We also tested whether disruption of the H-bond interaction between Q573 of the KQSKS loop and T581 side chain (Fig. 5a) could affect TM84 binding by mutating the threonine residue to a valine. This single T581V mutation, as well as the double mutation (Q573M/T581V) had no detectable effect on TM84 resistance in our bioassays (Supplementary Fig. 7a). However, addition of the T581V mutation to the AgnB2 Triple construct did produce an apparent increase in antibiotic sensitivity (Supplementary Fig. 7a).

### A miniloop substitutes for the LS domain in AgnB2 with implications for TM84-resistance and aminoacylation

Since AgnB2 lacks the LS domain and possesses a short peptide with a unique salt bridge between residues D567 and R571 (Fig. 5a), we tested if removal of the salt bridge had any effect on TM84 sensitivity. The salt bridge was disrupted using a number of mutations (D567N, R571E, M and Q), and tested using the bioassays. No significant change in resistance was detected for these mutants compared with wt AgnB2 (Supplementary Fig. 7b). An L577K mutation designed to compete with R571 (Fig. 5) for the salt bridge to the residue D567 produced a clearing zone in our bioassays (Supplementary Fig. 7b) but that is likely due to loss of stability of the enzyme. Taken together, these results indicate that disruption of the D567-R571 salt bridge that replaces the LS domain is not sufficient to cause resistance to TM84, but it may play still play an important structural or canonical catalytic role in AgnB2.

### Conversion of a TM84-resistant enzyme into a sensitive form by altering key tRNA interactions

To test whether an LS domain can have a substantial effect on the TM84 sensitivity of a LeuRS, we examined recombinant LeuRS *H. pylori* (LeuRS_Hp_), which lacks an LS domain (Fig. 4). Interestingly, we found using *in vitro* aminoacylation assays that the LeuRS_Hp_ was still significantly inhibited by TM84 (IC_50_=34±1.14 nM; Supplementary Fig. 8) although to a lesser degree than LeuRS_At_ (ref. 23). Interestingly, this enzyme also contains a neutral Q413 residue in a homologous position to R418 from LeuRS_EC_ is thought to be involved in positioning of the tRNA CCA 3′-end in the enzyme active site^25^,^38^. We took this one step further, by introducing all three core mutations implicated in TM84 resistance in AgnB2 into LeuRS_Hp_ (P40T, N100A and M573Q). However, our *in vitro* aminoacylation analysis revealed that this enzyme was inactive under the conditions used in our study (Supplementary Fig. 8).

**Figure 7:**
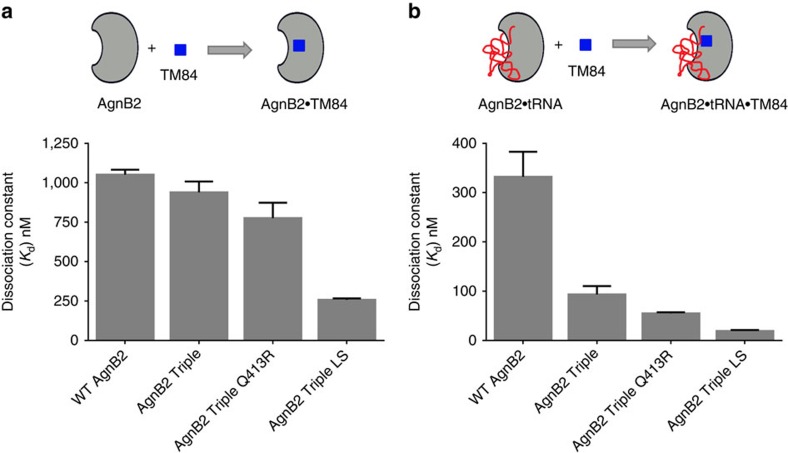
The accumulative mutation of tRNA interacting elements with core Triple mutations in AgnB2 increases the enzyme's affinity to TM84. ITC results depicted in graphical form compare the TM84-binding affinities of wt AgnB2 and Triple AgnB2 mutant±Q413R or LS domain to (**a**) TM84 alone; or (**b**) TM84 in the presence of tRNA^Leu^. Error bars represent standard deviation from three independent experiments.

To pursue further a possible role of the LS domain in TM84 sensitivity, we inserted the LS domain from LeuRS_At_ into AgnB2 LeuRS and tested the chimeric enzyme's sensitivity to TM84 in our bioassay. In addition, we also inserted the LS domain into the background of the AgnB2 Triple variant (T42P/A102N/Q573M). Our results show that insertion of the LS domain into AgnB2 wt had no effect on antibiotic sensitivity in our bioassays but when the LS domain was inserted into the AgnB2 triple background there was a substantial increase in clearing zone size (Fig. 6b). A comparison of the TM84 inhibition of aminoacylation activity of the AgnB2 Triple mutant + LS domain construct against wt AgnB2, at a fixed ATP concentration of 0.675 mM ATP (where no substrate inhibition of either enzyme is detected) showed a 34-fold (IC_50_ of 1.8 μM) decrease in the IC_50_ of the chimeric mutant compared with wild-type (IC_50_ of 61.3 μM) (Fig. 6c). ITC analysis of this construct was used to deconvolute the contribution of tRNA to the binding of TM84 to the mutant. Our results showed that TM84 binding alone (*K*_d_=258 nM) increased by fourfold (Fig. 7a) and TM84 binding in presence of tRNA (*K*_d_ =19 nM) increased by 17-fold respectively, compared with wt AgnB2 (Fig. 7b).

The positively charged R418 residue in LeuRS_Ec_ has been implicated in controlling the translocation of the negatively charged backbone of the tRNA^Leu^ CCA 3′-end into the synthetic active site^25^,^38^. We tested whether modification of the homologous position in AgnB2, Q413, from a neutral to positively charged arginine might modulate TM84 resistance through stabilization of the tRNA-dependent inhibition complex. Introduction of a single-point Q413R mutation into AgnB2 showed no effect on the size of clearing zones in our bioassays compared with wt (Fig. 6b). However, when we placed the Q413R mutation in the background of the AgnB2 Triple mutant (T42P/A102N/Q573M) there was a substantial increase in the clearing zone diameter suggesting an increase in TM84 sensitivity compared with the AgnB2 Triple (Fig. 6b). Analysis of the TM84 inhibition kinetics of the AgnB2 Triple + Q413R mutant showed an IC_50_ of 2.9 μM, 21-fold smaller than wt-AgnB2 at a fixed ATP concentration (Fig. 6c) indicating this construct had a significant increase in TM84 sensitivity compared with wt. We then analyzed the thermodynamics of binding of TM84 to the AgnB2 Triple Q413R·tRNA^Leu^ complex and found it was sixfold tighter than wt AgnB2 (Fig. 7b). However, the binding of TM84 to the AgnB2 Triple Q413R enzyme alone was only 1.3-fold tighter than the wt enzyme (Fig. 7a). This further suggested the importance of tRNA interacting residues for imparting sensitivity towards TM84.

In summary, our results suggest that the absence, or modification of, idiosyncratic tRNA binding elements in AgnB2, act cumulatively with critical core mutations on the periphery of the LeuRS active site, significantly contributing to the high degree of TM84 resistance observed in AgnB2.

## Discussion

TM84 (agrocin 84) is a highly potent LeuRS toxin that employs a unique tRNA-dependent inhibition mechanism. During production of agrocin 84, the biocontrol *A. radiobacter* strain 84 expresses the self-immunity LeuRS AgnB2 to prevent cell suicide. Our data show that AgnB2 achieves resistance to TM84 by minimizing the effect tRNA^Leu^ has on the toxin's affinity for the enzyme as well as weakening its affinity for tRNA^Leu^ and the inhibitor alone. Remarkably, this resistance phenotype is achieved without dramatically affecting the catalysis of the overall aminoacylation reaction (Table 1). Consistent with this observation, the X-ray crystal structure of the AgnB2·tRNA^Leu^·Leu-AMS ternary complex reveals that residues in the synthetic active site that directly bind Leu-AMP are not substantially different from other bacterial LeuRSs.

**Figure 8:**
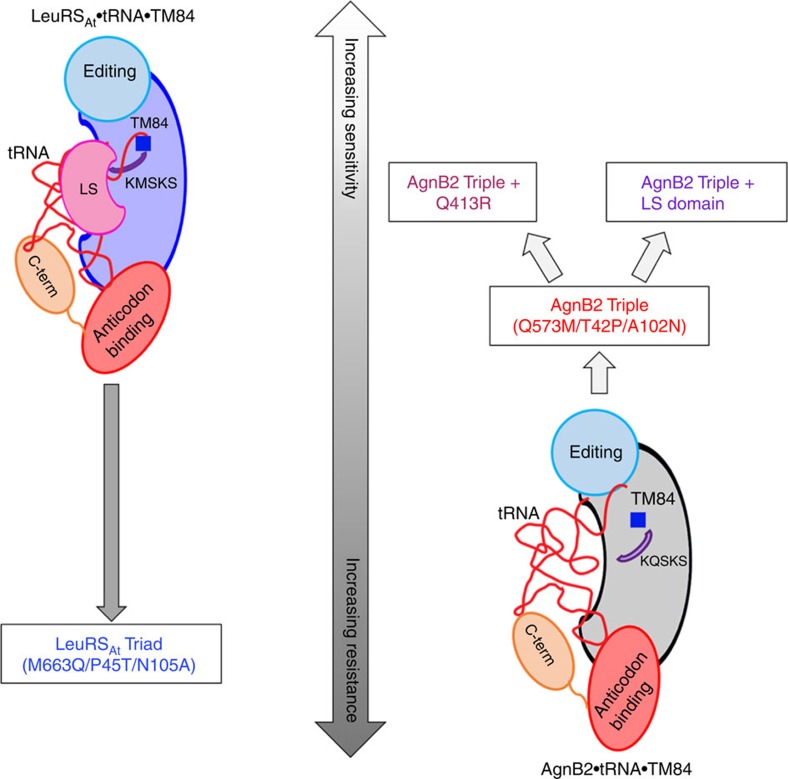
Model illustrating determinants of TM84 sensitivity and resistance in LeuRS_At_ and AgnB2. The ternary complex of LeuRS_At_ forms a tight binding complex with the CCA 3′-end of the tRNA and TM84. Upon mutation of three critical residues, P45, M633 and N105, a TM84 resistant mutant (LeuRS_At_ Triad) is obtained. AgnB2 on the other hand forms a weak ternary complex with tRNA and TM84, thereby explaining its high level of resistance towards TM84. Mutagenesis of the corresponding residues in AgnB2 with the residues in LeuRS_At_ results in the AgnB2 Triple mutant that has reduced levels of AgnB2 resistance. Upon introduction of Q413R mutation or addition of a LS-domain to the AgnB2 triple mutant, increased sensitivity towards TM84 is achieved. Thus these residues along with the LS domain may be the key determinants of sensitivity or resistance in LeuRS_At_ and AgnB2.

We identified key molecular determinants imparting TM84 resistance (or sensitivity) to LeuRSs by utilizing an approach based on the analysis of structures of the naturally resistant and sensitive enzymes AgnB2 and LeuRS_Ec_ (Fig. 8). LeuRS mutants that affected TM84 sensitivity were then tested using *in vitro* and *in vivo* approaches. Our data indicate that the molecular elements in LeuRSs that contribute to TM84 sensitivity or resistance are much more complex than one would expect *a priori*. Surprisingly, some of these determinants were not located directly in the active site but on the periphery (with distances ∼10–15 Å for most residues and 25 Å for Q413), including some residues interacting with the tRNA^Leu^. The core resistance mutations in the catalytic domain of TM84 resistant AgnB2 are located in KQSKS loop (Q573), just upstream of the HIGH loop (T42) or buttressing next to T42 in the case of A102. It is possible that the nature of the amino acids at these particular sites in LeuRSs may affect the molecular dynamics of these two critical catalytic motifs and ultimately altering the LeuRSs TM84 sensitivity phenotype. The exact contribution of other determinants to TM84 sensitivity at a greater distance from the active site is more difficult to discern. For AgnB2, alterations in tRNA-binding contacts, due to lack of an LS domain, C-term domain modifications, and the effect of Q413 on CCA 3′-end localization in the active site, may all play a role in destabilizing a TM84 ternary complex leading to resistance. It maybe that evolution has traversed a narrow energetic landscape towards a resistance phenotype by exploiting small energetic differences between mutants to produce larger scale changes in only certain kinetic and/or thermodynamic parameters. Presumably, there must be other additional unconserved residues that have played subtle compensatory roles on the pathway towards evolution of TM84 resistance.

Why did nature evolve TM84 to target LeuRS_At_ and not another AARSs from *A. tumefaciens*, and in particular the other Class I AARSs that have similar active site architectures such as IleRS and ValRS. TM84 does appear to be LeuRS specific, as the antibiotic does not inhibit agrobacterial IleRS^12^, and the single LeuRS *agnb2* gene is capable of providing agrocin 84-immunity to the pathogen. *In silico* docking analysis of TM84, from the LeuRS_Ec_ structure (PDB: 3ZGZ), into the catalytic domain of existing IleRS (PDB code: 1JZQ) and ValRS (PDB code: 1GAX) structures reveals steric clashes of the ‘leucine-like' region of TM84 with hydrophobic side chains in the active site (Supplementary Fig. 9). This analysis may provide some understanding of the LeuRS specificity of TM84. One possible reason that agrocin 84/TM84 evolved to target a LeuRS maybe because leucine is one of the most abundant amino acids in proteins, and so translation could be particularly sensitive to interference of LeuRS activity. A more plausible reason is that agrocin 84/TM84, or its evolutionary ancestor, had other roles in *Agrobacterium* biology that predisposed the toxin to evolve into a LeuRS inhibitor. The recent finding that the uptake molecule of agrocin 84, when cleaved from TM84, can act as signalling molecule binding to a key agrobacterial transcriptional regulator that controls quorum-sensing signal synthesis^9^ provides some support for this latter view.

Horizontal gene transfer of the *agnB2* gene on the pAgK84 plasmid from *A. radiobacter* strain K84 to the pathogen threatens emasculation of the biocontrol^5^,^16^,^17^. Genetic engineering of pAgK84 to prevent conjugative transfer of the plasmid to the pathogen has resulted in *A. radiobacter* K1026 strain^39^, the first bioengineered microbe approved for release into the environment^40^. Yet despite not containing any foreign DNA, this genetically altered strain is still only registered for use as a biopesticide in a limited number of countries (Gary Bullard and Allen Kerr, personal communications). In addition, as with all antibiotic interventions, continued success against resistance can never be completely assured. Thus, understanding the resistance mechanism of agrocin 84 immunity protein AgnB2 not only provides key insights into the mechanism of a fascinating tRNA-dependent toxin but also informs research into the continued crop protection provided by this biocontrol against the spectre of antibiotic resistance.

## Methods

### Generation of mutants of AgnB2 and LeuRS_At_

Mutations in AgnB2 and LeuRS_At_ were introduced using the Quikchange site-directed mutagenesis method (Stratagene). Mutagenesis was carried out in the *agnB2* gene previously cloned^12^ into the pET-28a vector (Novagen) for use in recombinant protein expression, and also in the *agnB2* gene subcloned into the broad host range plasmid pBBR1MCS-3 (ref. 41) for use in bioassay studies. Similarly, LeuRS_At,_ mutants were generated in the *A. tumefaciens leuS* gene cloned into the pET-21b vector (for recombinant protein expression) and pBBR1MCS-3 (bioassays). Multiple rounds of mutagenesis were performed to obtain more than one mutation in an enzyme. DNA sequence analysis was used to confirm the correct construction of the entire gene sequence for all single, and multiple site mutants, used in this study.

### Insertion of the LS domain of LeuRS_At_ into AgnB2

The LS domain of LeuRS_At_ (141 bp) was inserted into AgnB2 (replacing the region between amino acid no. 566 and 572 with the LS domain)^37^ To obtain this, the forward PCR primer was designed to contain the region of AgnB2 (∼25 bp) preceding the LS domain insertion followed by the first 25 bases of the LS domain (5′–3′). Similarly, the reverse primer was designed so as to include the last 25 bp of the LS domain (3′–5′) and the gene sequence of AgnB2 after the LS domain insertion. An extension reaction was carried out to obtain mega primers, (forward and reverse) encoding the entire LS domain (of LeuRS_At_) flanked by the sequence of AgnB2 on both sides using the protocol by Wang and Malcolm^42^. DpnI digestion was then carried out to remove the wt template. The resultant mega primer was gel purified using the Qiaex II gel extraction kit (Qiagen) to be used in the second step. The PCR amplified mega primers (generated in the first step) were then added to the extension reaction in presence of the pET-28a plasmid containing the *agnB2* gene (using the Quikchange site-directed mutagenesis protocol II). Positive clones encoding AgnB2 with the entire LS domain of LeuRS_At_ were identified by colony PCR and finally confirmed by DNA sequencing analysis. Using a similar strategy, LS domain was added to the AgnB2 Triple mutant as well as the AgnB2 Triple Q413R mutant.

### Construction of *A. tumefaciens* bioassay strains

A modified *acs* operon encoding the agrocin 84 transporter system (agrocinopine permease)^43^,^44^,^45^ was cloned into pYW15d plasmid containing a T5 promoter and an antibiotic marker (carbenicillin)^46^. Importantly, the *accR* repressor gene, responsible for the regulation of the agrocinopine permease^47^, had been removed from our construction in order to produce *A. tumefaciens* strains with maximum agrocin 84 uptake. The pYW15d-*acs ΔaccR* plasmid encoding the agrocin 84 transporter and the pBBR1MCS-3 plasmid^41^ containing the *agnB2*/*leuS*_*At*_ gene and a T7 promoter and a tetracycline marker were then transformed into an electrocompetent *A. tumefaciens* NTL4 T7*pol*-Gm-*FRT* strain containing the DE3 lysogen encoding a T7 RNA polymerase^48^. Briefly, electroporation was performed at 2400 V and 400Ω and the culture was then grown in 3 ml LB broth for 2.5 h. The *A. tumefaciens* culture was then streaked on LB agar plates containing 100 μg ml^−1^ of carbenicillin, 15 μg/ml of gentamycin and 10 μg/ml of tetracycline. The final construct *A. tumefaciens* NTL4 T7*pol*-Gm-*FRT* containing both pYW15d-*acs ΔaccR* (encoding the agrocin 84 transporter) and a pBBR1MCS-3 plasmid (containing a gene encoding either wt or mutant AgnB2/LeuRS_At_ proteins was then used for bioassay analysis. Individual colonies were picked and grown in 3 ml cultures to make glycerol stocks for storage at −80 °C.

### Protein expression and purification

All enzymes (mutants of AgnB2 and LeuRS_At_) were overexpressed in *E. coli* BL21 (DE3) RIL codon plus cells (Agilent) (containing pET-28a plasmid encoding C-terminal 6X His-tagged AgnB2 gene or pET-21b plasmid encoding C-terminal 6X histidine tagged LeuRS_At_ gene). LeuRS enzymes were purified in a similar manner as detailed previously for LeuRS_At_^23^. In brief, His-tagged proteins were purified by affinity chromatography using Ni-NTA agarose beads (Qiagen), followed by purification using a strong anion-exchange Mono-Q column (GE Healthcare) by FPLC. All proteins were confirmed to be ∼95% pure using SDS–polyacrylamide gel electrophoresis analysis and the active concentration determined by active-site titration.

### Purification of TM84

Agrocin 84 and TM84 were obtained from *A. tumefaciens* NT1 (pAgK84::Tn5A1-B5) agrocin 84 secretion strain grown at 28 °C in D-glucose minimal media supplemented with 50 μg ml^−1^ kanamycin as described previously^9^,^23^. Briefly, agrocin 84 and TM84 was separated from other media components by adsorption on charcoal, and then eluted using 70–90% reagent alcohol, dried and both further purified by reverse-phase HPLC. The concentration of TM84 was determined using an extinction coefficient (*ɛ*=260 nm) of 0.0154 M^−1^ cm^−1^.

### Bioassays

*In vivo* assays to determine the agrocin 84 sensitivity of AgnB2/LeuRS_At_ and their mutants were performed. Stationary phase cultures of *A. tumefaciens* NTL4 T7*pol*-Gm-*FRT* strains transformed with both pYW15d-*acs ΔaccR* (encoding the agrocin 84 transporter) and the pBBR1MCS-3 plasmids (containing genes encoding the wt or mutant AgnB2/LeuRS_At_ proteins) were first diluted 1:100 and then added to 20 ml LB agar plates containing 100 μg/ml of carbenicillin, 15 μg ml^−1^ of gentamycin, 10 μg ml^−1^ of tetracycline and 1mM IPTG. A well was punched in the centre of the plate and 50 μl of 60 μM agrocin 84 was added to determine the sensitivity of agrocin 84 towards AgnB2 and LeuRS_At_. The plates were then incubated at 28 °C for 48 h. The sensitivity of the strain towards agrocin 84 was determined by measuring the zone of clearance around the well containing agrocin 84. The control strain containing only the *acs* operon, but no pBBR1MCS-3 plasmid showed a zone of clearance of around 4 cm. Therefore, any sample having a zone of clearance >4.1 cm was considered inactive.

### tRNA^Leu^ transcription

In this study, we used purified *in vitro* transcribed tRNA^Leu^(UAA) isoacceptor based on the nucleotide sequence from *A. tumefaciens* (referred to in text as agrobacterium tRNA^Leu^) and not *A. radiobacter*. This allowed a direct comparison of *in vitro* aminoacylation kinetics and ITC analysis with our *in vivo* studies of AgnB2 mutants. Importantly, both tRNA^Leu^ isoacceptors from both species share a substantial similarity. Agrobacterium tRNA^Leu^ with (UAA) isoacceptor was obtained by treating plasmids encoding the tRNA^Leu^ gene with *Bst*NI overnight at 60 °C. The linearized DNA was then used as a template in the *in vitro* transcription reaction containing 40 mM Tris HCl (pH 8.0), 25 mM MgCl_2_, 40 mM DTT, 0.1% Triton X-100, 1 mM spermidine and 2 mM rNTPs, 1 U ml^−1^ RNase inhibitor and 9 μM T7 RNA polymerase^23^. After DNase I treatment for removal of template DNA and quenching the reaction with EDTA, the tRNA was precipitated using 0.3 M sodium acetate and absolute ethanol. The tRNA obtained was further purified on a 12% denaturing PAGE gel (19:1) containing 8 M urea and 1X TBE buffer (pH 8.3). Gel extraction of the tRNA was then performed, followed by refolding of tRNA at room temperature using 1 mM MgCl_2_ after incubating 1 min at 80 °C (refs 23, 49). The concentration of tRNA was determined and corrected by a factor of 1.34 to account for the hypochromic effect on absorbance^23^.

### Active-site titration assay

Active enzyme concentrations of AgnB2, LeuRS_At_ and their respective mutants was determined using the method described by Beebe *et al*.^50^ and Ferst^51^. In brief, purified enzymes in the range of 1-2 μM (as determined by the Bio-Rad Protein assay) were added to a reaction mix containing 20 mM KCl, 10 mM MgCl_2_, 10 mM β-mercaptoethanol (β-ME), 1 mM L-leucine, 5 μg ml^−1^ yeast inorganic pyrophophatase (NEB), 5 μM ATP and 4 μCi [γ-32P] ATP. The reaction was initiated at 28 °C and 5 μl aliquots of the reaction quenched using 7% perchloric acid and 1 M HCl over a time period of 5–30 min. The amplitude of the burst phase of Leu-AMP formation was used to determine the amount of Leu-AMP formed and subsequently the active concentration of the enzymes.

### ATP/PPi exchange assay

The formation of Leu-AMP by ATP/PPi exchange assays was performed as described earlier^23^,^50^. Briefly, the reaction buffer contained 50 mM HEPES buffer (pH 7.4), 20 mM KCl, 10 mM MgCl2, 500 μM L-leucine, 4 mM ATP, 1 mM tetrasodium pyrophosphate (NaPPi), 10 μCi/mmol [^32^P] NaPPi and 1 μM bovine serum albumin (BSA). The reaction was initiated by addition of 1–4 nM of active enzyme and incubated at 28 °C. Aliquots (5 μl each) were collected over 30 min and quenched in a 10% charcoal slurry containing 0.5% HCl and 50 μl of 200 mM sodium pyrophosphate in 1 M HCl. Liquid scintillation counting was then used to measure the amount of [^32^P]-PPi incorporation into ATP that was adsorbed onto the charcoal.

### Aminoacylation assay

Aminoacylation activities of AgnB2, LeuRS_At_ and their mutants were measured in 50 mM HEPES buffer (pH 7.4) containing 20 mM KCl, 25 mM MgCl_2_, 25 mM β-ME, 4 mM ATP, 500 μM [3,4,5-^3^H] L-leucine (20 μCi/mmole), 5 μg/ml inorganic pyrophosphatase, 1 μM BSA, 10 μM of active *in vitro* transcribed tRNA^Leu^ and enzymes in the range of 2–8 nM. Reactions were pre-incubated with or without substrate and/or inhibitor and then carried out at 28 °C (37 °C-LeuRS_Hp_). Aliquots of the reaction (5–10 μl) were quenched on 3 mM filter pads (Whatman, GE Healthcare) presoaked with 5% trichloroacetic acid (TCA). The pads were washed thrice with 5% TCA and 90% ethanol at 4 °C and then dried. The amount of Leu-tRNA^Leu^ was then quantified using scintillation counting. Each reaction was performed in triplicate and analyzed using Kaleidagraph (for *K*_m_ and *k*_cat_ determinations) or GraphPad Prism software (for IC_50_ determination).

### Isothermal titration calorimetry

The affinity of TM84 binding to AgnB2, LeuRS_At_ and their mutants was determined using ITC as described earlier^23^. Binary interactions (TM84 binding to protein) and ternary interactions (TM84 binding to protein+tRNA (1:1.2 molar ratio)) were tested using an Auto-ITC200 microcalorimeter (from MicroCal/GE Healthcare). Typically, about 4–8 μM active protein solution and a 10-fold higher ligand concentration were prepared in buffer containing 50 mM HEPES (pH 7.4), 20 mM KCl 10 mM MgCl_2_ and 1 mM β-ME. The titrations were carried out in triplicate at 28 °C. The data was then analyzed using Origin software (version 7) and fitted to a one-site or a two-site binding model to obtain the binding affinity (*K*_d_), stoichiometry (N) and enthalpy (ΔH) for each complex.

### Crystallization

Crystallization was carried out at 20 °C by the hanging drop vapor diffusion method. Crystals of the ternary complex AgnB2 LeuRS·tRNA^Leu^·Leu-AMS were obtained by mixing 1 μl of a solution containing AgnB2 LeuRS at 43 μM, tRNA^Leu^ (*A. tumefaciens*) at 34 μM and Leu-AMS at 2 mM; with 1 μl of reservoir solution containing 0.1 M MES (pH 6.5), 0.2 M ammonium sulfate and 27% (w/v) PEG 5000 MME. Quality and size of crystals were optimized by macro-seeding with protein at 2 mg ml^−1^. The crystals were frozen in liquid nitrogen after transfer for a few seconds in the mother liquor plus 15% (v/v) ethylene glycol as cryoprotectant.

### Structure determination and refinement

The diffraction data sets of the complex *AgnB2* LeuRS-tRNA^Leu^-Leu-AMS were collected at the European Synchrotron Radiation Facility (ESRF, France). The data sets were integrated and scaled with the XDS suite^52^. Further data analysis was performed with the CCP4 suite^53^. The structure was initially solved by molecular replacement with PHASER^54^ using as a model the catalytic and anticodon-binding domains of a model built with SWISS-Model^55^ based on homology to LeuRS_Tt_ (PDB 2BTE)^56^ and LeuRS_Ec_ (PDB: 4AS1)^25^. The molecular replacement solution was used to search with PHASER for AgnB2 tRNA^Leu^ using as a model the core of *E. coli* tRNA^Leu^ (bases 1–73, except the bases belonging to the long-variable arm). The obtained model was completed by manual placing of the ZN1 domain, leucine-specific domain, C-terminal domain and editing domain. Manual adjustments were done with COOT^57^ and the final model was refined using REFMAC5 (ref. 58). Structure quality was analysed with MOLPROBITY (http://molprobity.biochem.duke.edu/) and showed all residues in allowed regions. Figures were drawn with PYMOL (http://www.pymol.org/).

### Molecular modelling

A homology model of LeuRS_At_ was built using the Insight II Molecular Modeling System (www.accelrys.com) based upon the template structure of the LeuRS_Tt_ (PDB ID 1H3N)^35^. A normalized sequence-structure compatibility score of 0.77 was calculated for the LeuRS_At_ homology model using the Verify the three-dimensional module of Insight II^59^,^60^.

### Data availability

Atomic coordinates and structure factors for the AgnB2·tRNA·Leu-AMS ternary complex have been deposited in the RCSB Protein Data Bank with the accession code 5AH5. All other data associated with this manuscript are available from the corresponding author on reasonable request.

## Additional information

**How to cite this article:** Chopra, S. *et al*. Structural characterization of antibiotic self-immunity tRNA synthetase in plant tumour biocontrol agent. *Nat. Commun.* **7,** 12928 doi: 10.1038/ncomms12928 (2016).

## Supplementary Material

Supplementary Information Supplementary Figures 1-9, Supplementary Tables 1-2 and Supplementary References.

## Figures and Tables

**Figure 1:**
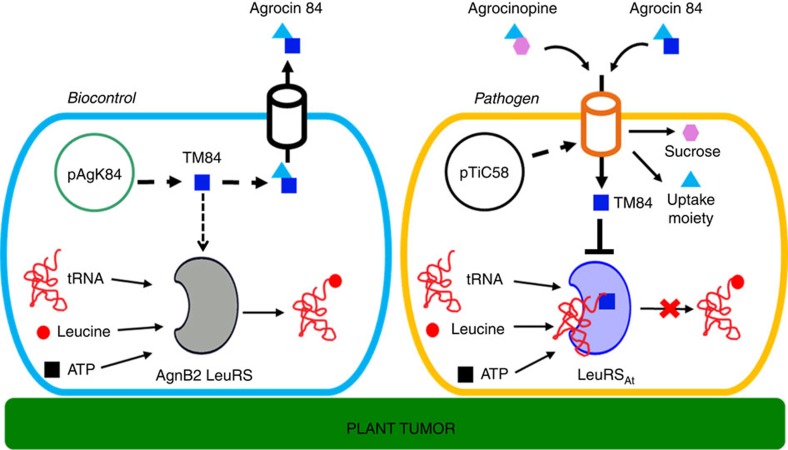
Biology of agrocin 84 produced by biocontrol *A. radiobacter* strain K84. Pathogenic *A. tumefaciens* possesses a TiC58 plasmid that upon infection of the host plant makes the plant produce a carbon and phosphate source agrocinopine. The pathogen takes up agrocinopine via an agrocinopine permease encoded on pTiC58. This transporter also recognizes the uptake moiety on agrocin 84, an antibiotic produced by the plant tumour biocontrol, *A. radiobacter* strain K84. Upon entry of agrocin 84 into the pathogen, it is cleaved into the toxic moiety TM84 and the transport moiety. TM84 targets the leucyl tRNA synthetase, thereby inhibiting the aminoacylation of tRNA^Leu^. This subsequently leads to the cessation of protein synthesis in the pathogen and leads to cell death. TM84 however has no effect on the aminoacylation reaction of a self-immunity LeuRS called AgnB2 that is encoded by the pAgK84 plasmid in *A. radiobacter* K84.

**Table 1:** Kinetic parameters obtained for the aminoacylation and ATP-PPi exchange reactions catalysed by LeuRS_At_ and AgnB2 at 28 °C at pH 7.4.

**Enzyme**	**Substrate**	***K***_**m**_ **(μM)**	***k***_**cat**_ **(s**^**−1**^**)**
*Aminoacylation*			
AgnB2	ATP	95.4±18.3	0.43±0.02
	Leucine	28.7±7.3	0.35±0.03
	tRNA	1.48±0.25	0.43±0.02
LeuRS_AT_*	ATP	396±83	0.33±0.02
	Leucine	38±3.2	0.38±0.01
	tRNA	0.94±0.19	0.36±0.02
			
*ATP-PP*_*i*_ *Exchange*			
AgnB2	ATP	1047±140	67±8.9
	Leucine	12±2	73±3.6
LeuRS_AT_	ATP	357±22	87±1.5
	Leucine	9.7±0.7	85±1.6

All data in this table were average values obtained from three independent experiments with error±values representing s.d.

^*^From reference Chopra, *et al*.^23^.

**Table 2:** Data collection and refinement statistics

***Agrobacterium radiobacter strain K84*****AgnB2 LeuRS+tRNA**^**Leu**^**+Leu-AMS**	
*Data collection*	
Space group	*P*2_1_
Cell dimensions	
*a*, *b*, *c* (Å)	169.91, 50.32, 170.52
*α*, *β*, *γ* (°)	90.00, 93.48, 90.00
Resolution (Å)*	50-2.1 (2.10-2.20)
*R*_sym_	7.5 (65.2)
*I*/*σI*	10.6 (2.0)
Completeness (%)	99.5 (99.8)
Redundancy	3.7 (3.7)
	
*Refinement*	
Resolution (Å)	48.6-2.1 (2.15-2.10)
No. of reflections work/free	160,330/8,487
*R*_work_/*R*_free_	0.194 (0.309)/0.237 (0.331)
*No. of atoms*	
Protein	[6,307]^A^, [6,141]^B^
tRNA^Leu^	[1,702]^C^, [1,721]^D^
Ligand	62 [2 × Leu-AMS]
Water/SO_4_^−2^	871/25 [5 × SO_4_^−2^]
Mn^2+^	2
*B*-factors	
Protein	[52.3]^A^, [48.6]^B^
tRNA^Leu^	[57.7]^C^, [54.9]^D^
Ligand	[27.8]^E^, [26.6]^F^
Water/SO_4_^−2^	44.4/86.2
Mn^2+^	87.3
RMS deviations	
Bond lengths (Å)	0.016
Bond angles (°)	1.736

Values in brackets correspond to averages of the corresponding chain, indicated as superscript.

^*^Values in parentheses are for the highest-resolution shell.
