# Supplementary material for: Structural characterization of antibiotic self-immunity tRNA synthetase in plant tumour biocontrol agent
Source: Nat Commun. 2016 Oct 7;7:12928. doi: 10.1038/ncomms12928 (PMC5059758; doi:10.1038/ncomms12928)
Supplement: Supplementary Information — Supplementary Figures 1-9, Supplementary Tables 1-2 and Supplementary References. [file ncomms12928-s1.pdf]

Supplementary Figures

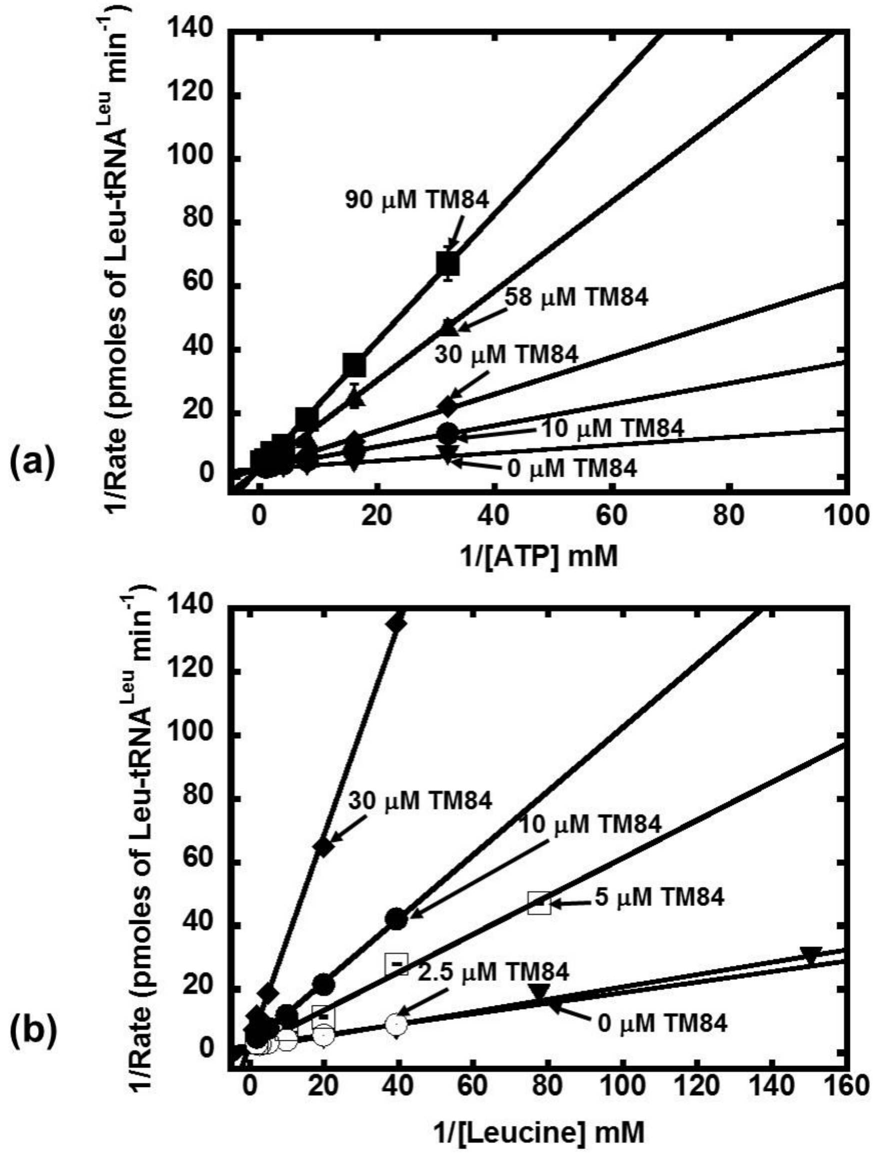

Supplementary Figure 1. TM84 is a weak inhibitor of AgnB2 and competitive with both ATP (a) and leucine (b) binding to the active site. Lineweaver-Burk plots of the inhibitory effects of TM84 on the aminoacylation activity of wt AgnB2 in the presence of a) varying ATP concentrations.with [TM84] inhibitor at 0 μM (▼), 10 μM (●), 30 μM (◆), 58 μM (▲) and 90 μM (■), and a fixed 500 μM leucine

concentration; and b) varying leucine concentrations and [TM84] inhibitor at 0 μM (▼), 2.5 μM (○),5 μM (□), 10 μM (●) and 30 μM (◆) and a fixed ATP concentration of 1 mM. The reactions were carried out using 2 nM enzyme at 28 °C, pH 7.4 The apparent K_i for TM84 varying ATP concentration (4.72 ± 0.75 μM) and apparent K_i for TM84 varying leucine concentration (1.38 ± 0.28 μM) was determined from non-linear fits of duplicate data sets using the Graphpad Prism software.

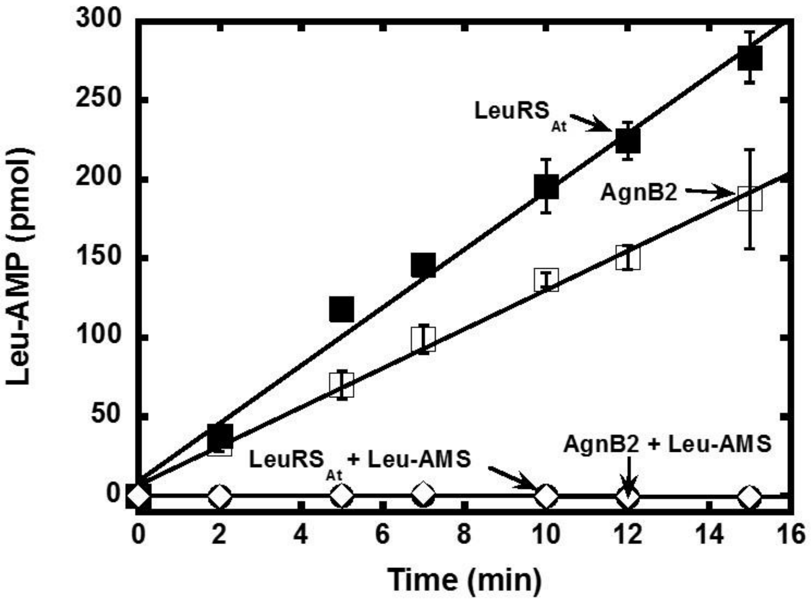

Supplementary Figure 2. Leu-AMS potently inhibits Leu-AMP formation by both AgnB2 and LeuRS$_{At}$. ATP-PP$_i$ exchange reactions of LeuRS$_{At}$ (■) and wt AgnB2 (□) and the effect of 25 nM Leu-AMS on the production of Leu-AMP by LeuRS$_{At}$ (●) and wt AgnB2 (○). Three independent reactions were carried out using 1 nM enzyme at 28 °C, pH 7.4.

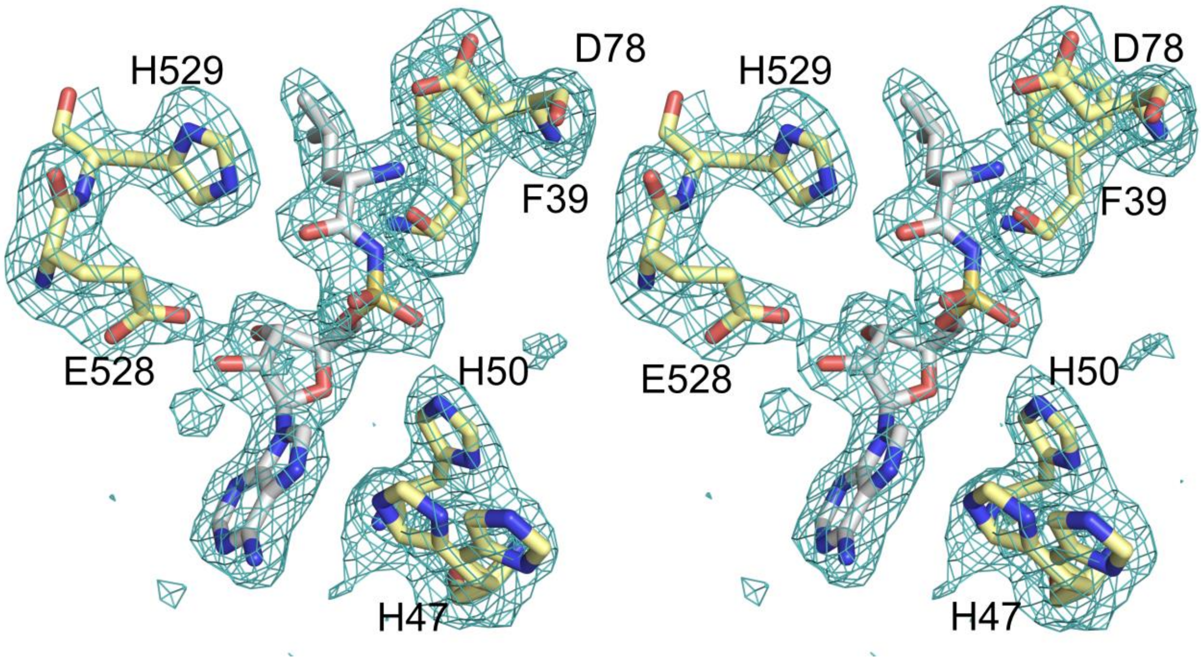

Supplementary Figure 3. Unbiased electron density difference map. Stereo diagram showing unbiased m(Fo-Fc) difference electron density (contoured at 3.0 σ) for leucyl-adenylate analogue (Leu-AMS) bound into the editing site of AgnB2. Key residues of the LeuRS catalytic site are shown in yellow and Leu-AMS in white sticks.

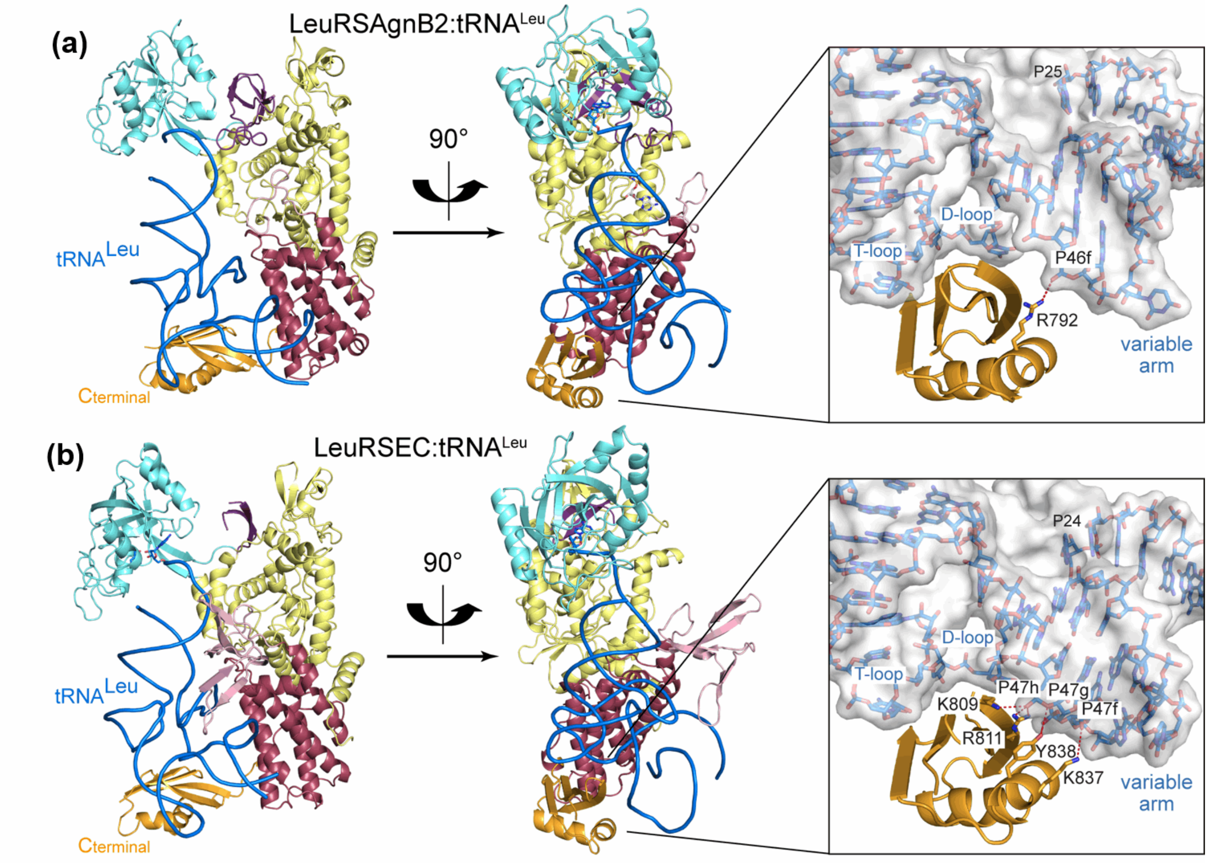

Supplementary Figure 4. Agrobacterial tRNA^{Leu} makes fewer interactions with the C-terminal domain of AgnB2 as compared to the interactions of *E. coli* tRNA^{Leu} with LeuRS$_{Ec}$. a) The short variable arm of *A. tumefaciens* tRNA^{Leu} forms interactions with the C-terminal domain at base 46f in the AgnB2•tRNA^{Leu}•Leu-AMS complex. b) *E. coli* tRNA^{Leu} possesses a longer variable arm resulting in increased interactions at bases 47f-47i with the C-terminal domain in the LeuRS$_{Ec}$•tRNA^{Leu}•Leu-AMS structure [1].

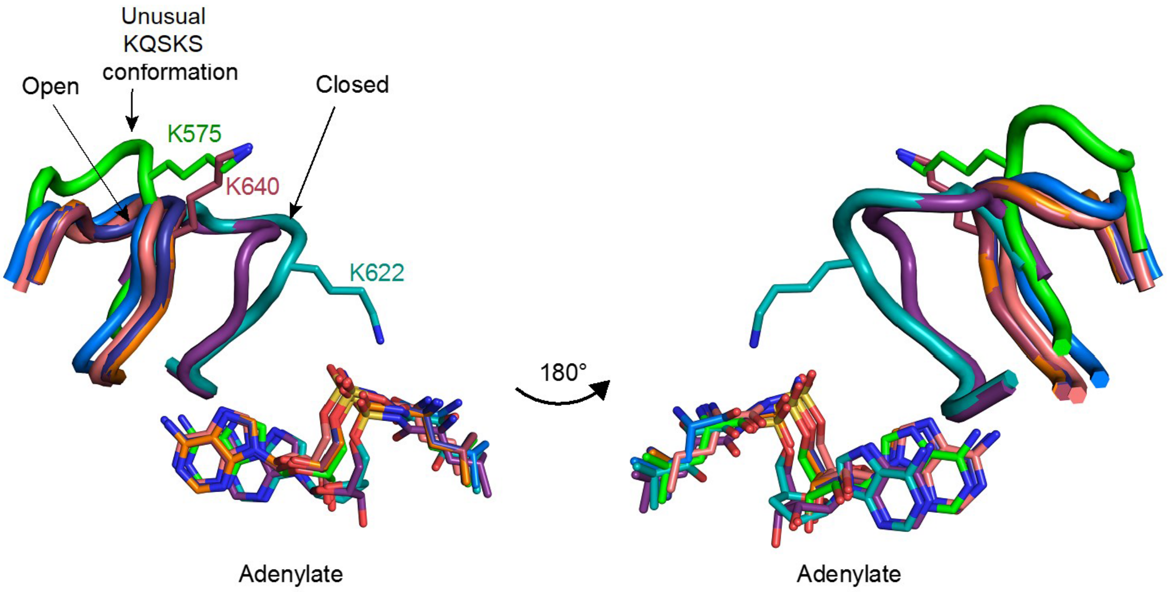

Supplementary Figure 5. Superimposition of AgnB2 onto LeuRS$_{Tt}$ and LeuRS$_{Ec}$ structures in different conformations to compare positioning of the KM(Q)SKS loops. The structure of AgnB2•tRNA^{Leu}•Leu-AMS complex (green) was superimposed onto the following LeuRS structures: LeuRS$_{Ec}$•tRNA^{Leu}•TM84 (teal-'aminoacylation-like' conformation, PDB-3ZGZ); LeuRS$_{Ec}$•tRNA^{Leu}•Leu-AMS (violetpurple-aminoacylation conformation, PDB-4AQ7); LeuRS$_{Ec}$•tRNA^{Leu} (yellow-editing conformation, PDB-4ARC); LeuRS$_{Tt}$•Leu-AMS (raspberry, PDB-1H3N); LeuRSTt•tRNALeu•AN2690 in editing domain (dark orange editing conformation, PDB-2V0C); LeuRS$_{Tt}$•tRNA^{Leu}•postransfer editing substrate (blue editing conformation, PDB-2BTE); LeuRS$_{Tt}$•posttransfer editing substrate analog (purple-blue, PDB-1OBC);and LeuRS$_{Tt}$•pre-transfer editing substrate analog (deepsalmon, PDB-1OBH). The two images (views 90° apart) show KM(Q)SKS loop regions and overlayed ligands in active site. Structural alignment was achieved by overlapping the α-carbons of LeuRS catalytic domains.

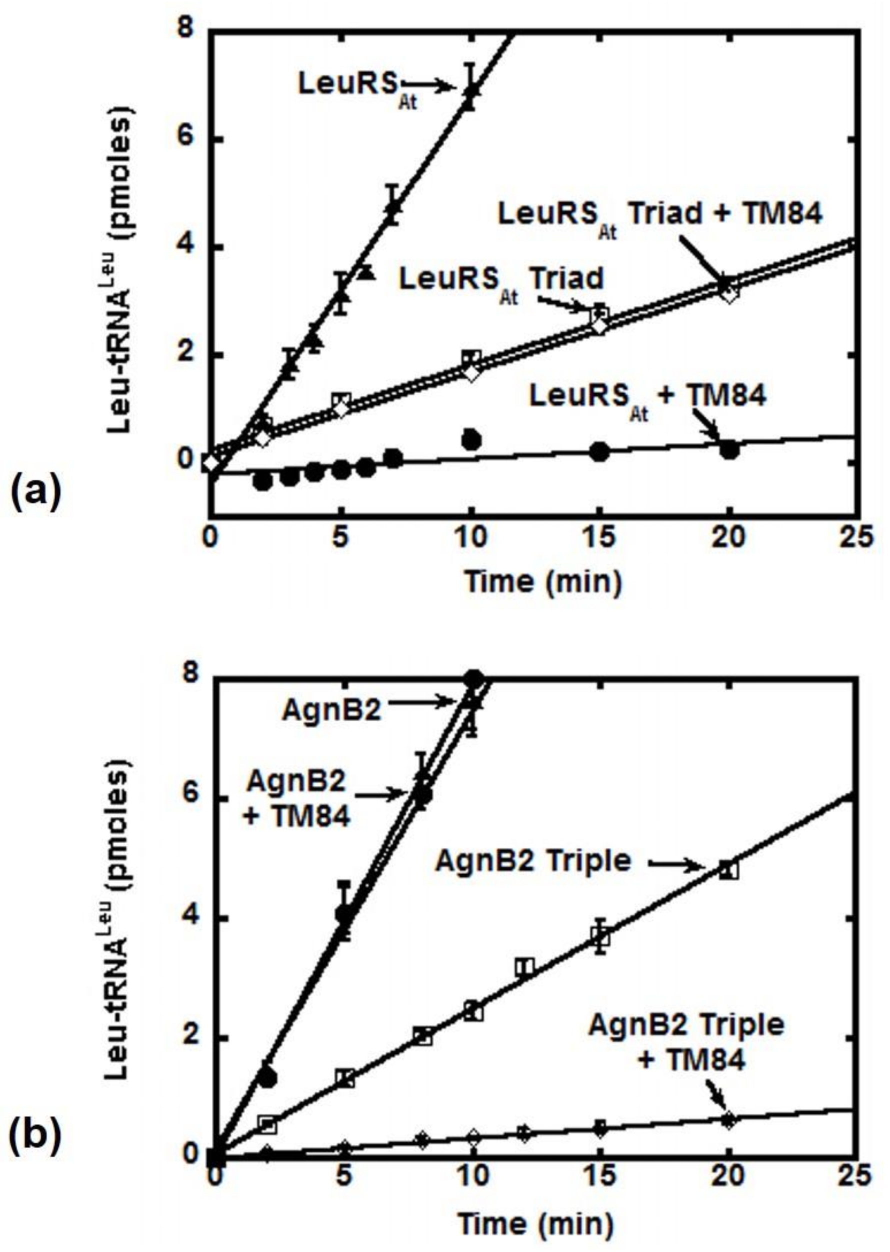

Supplementary Figure 6. Comparison of the effect of reciprocal core mutations in the catalytic core of $\mathbf{LeuRS_{At}}$ and AgnB2 on TM84 sensitivity. Initial rate aminoacylation reactions of a) Triad $LeuRS_{At}$ in comparison with wt $LeuRS_{At}$ in the presence and absence of TM84. In the presence of 20 nM TM84 only 4% activity remained for wt $LeuRS_{At}$ whereas TM84 concentrations as high as 500 nM had almost no effect on the Triad $LeuRS_{At}$ activity**;** and b) AgnB2 Triple aminoacylation rate in comparison with wt AgnB2 in the presence and absence of TM84. No change

in aminoacylation activity was observed for wt AgnB2 even at 10 μM TM84, while AgnB2 Triple showed 13% activity remaining in the presence of 10 μM TM84. All reactions were carried out in triplicate in the presence of 4 nM enzyme and 0.63 mM ATP at 28 °C, pH 7.4.

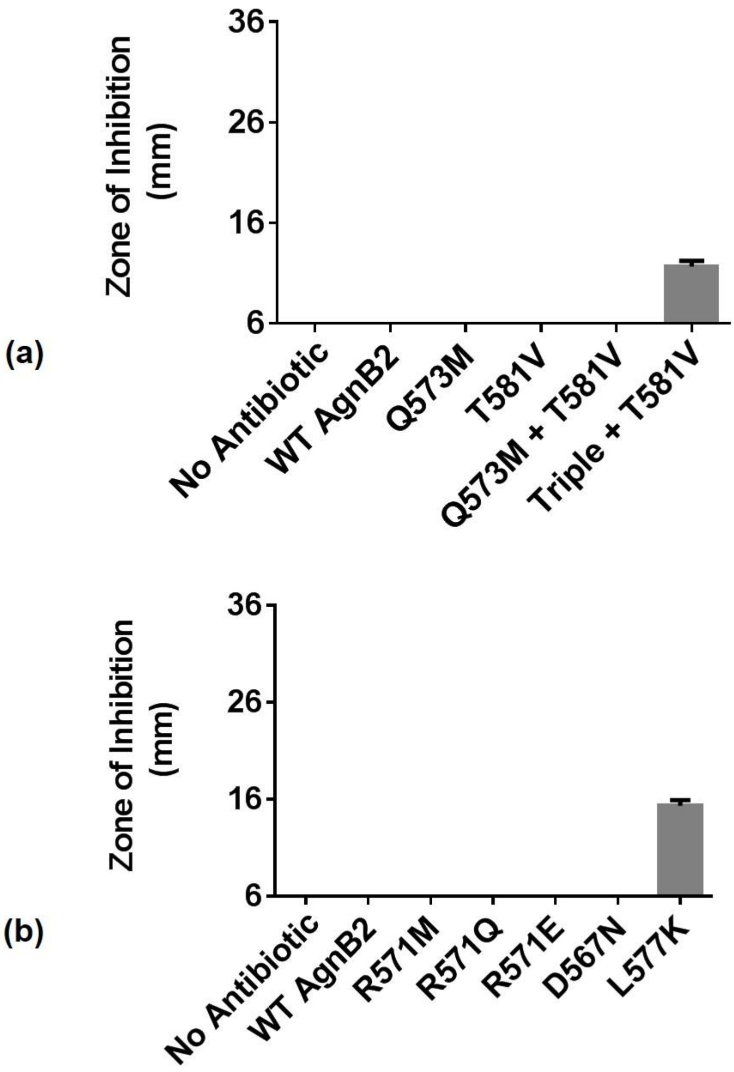

Supplementary Figure 7. Comparison of agrocin 84 sensitivity for additional AgnB2 mutants. Agrocin 84 bioassay results in graphical results for additional AgnB2 mutants mentioned in text. The clearing zones of lawns of *A. tumefaciens* NTL4 T7*pol*-Gm-*FRT* (pYW15d-*acs* Δ*accR)* strains containing plasmids encoding AgnB2 mutants with central wells each containing 50 μl of agrocin 84 [60 μM] were measured as detailed in Methods. Each bioassay experiment was carried out in triplicate with error bars representing standard deviation.

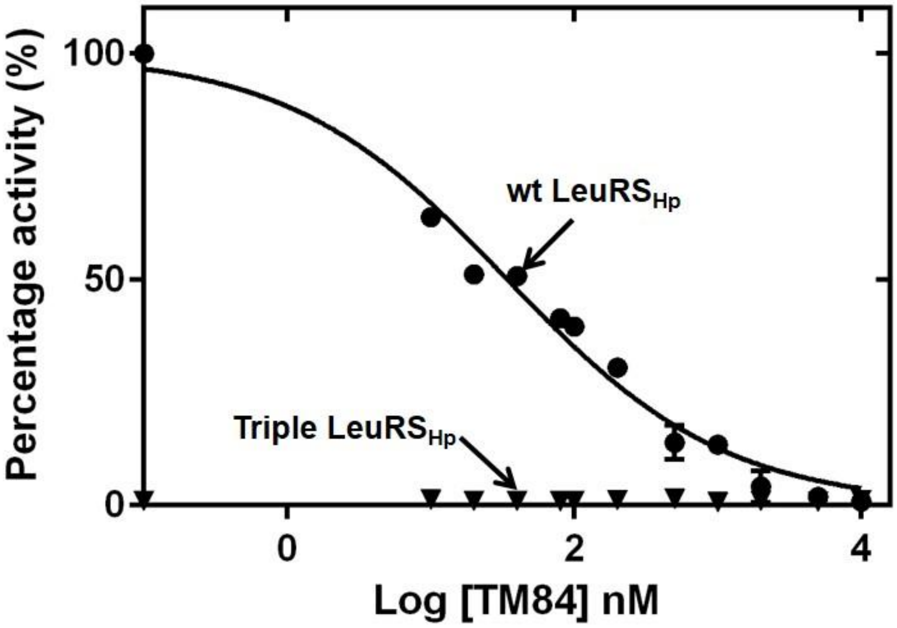

Supplementary Figure 8. Inhibition of wt LeuRS$_{HP}$ by TM84. TM84 dose-response curve of (●) wt LeuRS$_{Hp}$ and (▼) LeuRS$_{Hp}$ Triple catalysed aminoacylation of *agrobacterium* tRNA^{Leu}. Each reaction was carried out at 37 °C in using 4 nM of each enzyme and at varying concentrations of TM84. An IC_{50} value of 34 ± 1.14 nM was determined for wt LeuRS$_{Hp}$ using a non-linear fit of the above data using the Graphpad Prism software. No activity was detected for LeuRS$_{Hp}$ Triple under these conditions. Error bars represent standard deviation (n=3).

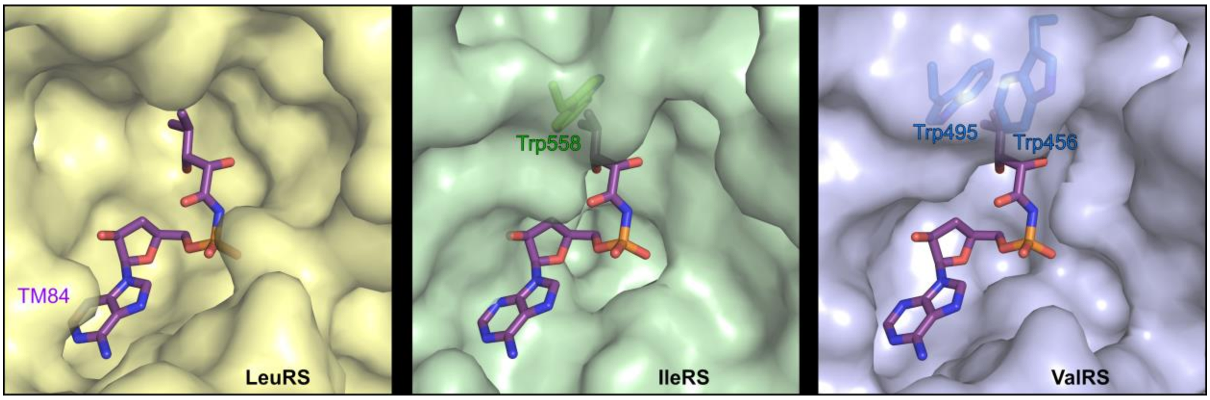

Supplementary Figure 9. TM84 specificity for LeuRS. (a) TM84 bound into the catalytic site of LeuRS (PDB code: 3ZGZ), with TM84 shown in violet sticks and protein surface in yellow. **(b)** Docking of TM84 into the catalytic site of IleRS (PDB code: 1JZQ), with protein surface shown in green and residues clashing to the 'leucine-like' moiety of TM84 shown in green sticks. **(c)** Docking of TM84 into the catalytic site of ValRS (PDB code: 1GAX), with protein surface in light blue and residues clashing to TM84 shown in blue sticks. Dockings of TM84 into IleRS and ValRS were performed by structural overlapping of the main chain atoms of their catalytic sites to the catalytic site of LeuRS (PDB code: 3ZGZ).

Supplementary Tables

Protein/Complex + Ligand	Model	K_d (nM)	Stoichiometry	ΔH (kcal mol^{-1})	-TΔS (kcal mol^{-1})	ΔG (kcal mol^{-1})
wt AgnB2 + Leu-AMS	One site	4.4 ± 0.43	0.96 ± 0.06	-12.8 ± 2.7	1.52 ± 0.5	-11.5 ± 0.07
wt AgnB2 + TM84	One site	1052 ± 31	1.08 ± 0.04	-8.4 ± 0.1	0.12 ± 0.1	-8.2 ± 0.02
wt AgnB2 • $tRNA^{Leu}$ + TM84	One site	332 ± 51	0.96 ± 0.1	-10.9 ± 0.6	1.90 ± 0.7	-9.0 ± 0.09
wt AgnB2 + $tRNA^{Leu}$	One site	310 ± 23	0.82 ± 0.02	-22.8 ± 2.3	13.8 ± 2.3	-9.0 ± 0.05
AgnB2 Triple + TM84	One site	940 ± 68	1.13 ± 0.05	-12.5 ± 0.4	4.1 ± 0.5	-8.3 ± 0.10
AgnB2 Triple • $tRNA^{Leu}$ + TM84	One site	93.4 ± 17	1.05 ± 0.10	-15.6 ± 0.5	5.9 ± 0.5	-9.7 ± 0.09
AgnB2 Triple + $tRNA^{Leu}$	One site	469 ± 30	1.1 ± 0.07	-12.6 ± 3.3	3.05 ± 3.6	-9.5 ± 0.41
AgnB2 Triple LS + TM84	One site	258 ± 8.75	0.9 ± 0.09	-47.9 ± 4.5	38.8 ± 4.4	-9.07 ± 0.09
AgnB2Triple LS • $tRNA^{Leu}$ + TM84	One site	19.2 ± 2.05	0.97 ± 0.08	-31.0 ± 0.7	20.4 ± 0.7	-10.6 ± 0.07
AgnB2 Triple Q413R + TM84	One site	776 ± 97	0.97±0.10	-11.9±1.12	-11.6 ±2.4	-8.42 ± 0.08
AgnB2 Triple Q413R • $tRNA^{Leu}$ + TM84	One site	55 ± 2.13	1 ± 0.01	-13.6 ± 0.05	-3.68 ± 0.001	-9.97 ± 0.04

Supplementary Table 1. ITC data showing binding of Leu-AMS, TM84 and/or $tRNA^{Leu}$ to wt AgnB2 and its mutants. All data in this table are average values obtained from 3 independent determinations with ± errors representing the standard deviation.

Protein/Complex + Ligand	Model	K_d (nM)	Stoichiometry	ΔH (kcal mol^{-1})	-TΔS (kcal mol^{-1})	ΔG (kcal mol^{-1})
*wt $LeuRS_{At}$ + Leu-AMS	One site	1.67 ± 0.86	1.04 ± 0.07	-23.3 ± 1.10	10.9 ± 1.3	-12.4 ± 0.48
*wt $LeuRS_{At}$ + TM84	One site	152 ± 20	0.93 ± 0.08	-10.4 ± 0.3	0.95 ± 0.4	-9.4 ± 0.08
*wt $LeuRS_{At}$ • $tRNA^{Leu}$ +TM84	Two site	0.81 ± 0.31<br>14.4 ± 1.37	0.51 ± 0.03<br>0.60 ± 0.01	-18.8 ± 0.9<br>-27.8 ± 0.7	6.2 ± 0.5<br>16.3 ± 0.5	-12.6 ± 0.2<br>-10.8 ± 0.01
*wt $LeuRS_{At}$ + $tRNA^{Leu}$	One site	84.9 ± 6.1	0.93 ± 0.01	-16.2 ± 0.6	6.4 ± 0.6	-9.7 ± 0.04
$LeuRS_{At}$ Triad +· Leu-AMS	One site	3.41 ± 1.8	1.16 ± 0.05	-41.4 ± 0.8	30.0 ± 0.8	-11.6 ± 0.8
$LeuRS_{At}$ Triad + TM84	One site	1552 ± 212	1.16 ± 0.06	-15.0 ± 0.9	7.0 ± 1.0	-8.0 ± 0.08
$LeuRS_{At}$ Triad • $tRNA^{Leu}$ + TM84	Two site	23 ± 14<br>366 ± 23.6	0.4 ± 0.04<br>0.59±0.08	-33.7 ± 1.9<br>-50.4 ± 4.7	23.0 ± 1.8<br>41.5 ± 4.7	-10.7 ± 0.3<br>-8.8 ± 0.06
$LeuRS_{At}$ Triad + $tRNA^{Leu}$	One site	211 ± 10	0.27 ± 0.14	-30.2 ± 2.6	21.0 ± 2.6	-9.2 ± 0.02

**Chopra et al.*, (2013) [2]

Supplementary Table 2. Isothermal titration calorimetry data showing binding of Leu-AMS, TM84 and/or $tRNA^{Leu}$ to $LeuRS_{At}$ and $LeuRS_{At}$ Triad mutant. All data in this table are average values obtained from 3 independent determinations with ± errors representing the standard deviation.

Supplementary References

1. Palencia, A. et al. Structural dynamics of the aminoacylation and proofreading functional cycle of bacterial leucyl-tRNA synthetase. *Nat Struct Mol Biol* **19**, 677-84 (2012).

2. Chopra, S. et al. Plant tumour biocontrol agent employs a tRNA-dependent mechanism to inhibit leucyl-tRNA synthetase. *Nat Commun* **4**, 1417 (2013).
